# Amniotic epithelial Cell microvesicles uptake inhibits PBMCs and Jurkat cells activation by inducing mitochondria-dependent apoptosis

**DOI:** 10.1016/j.isci.2025.111830

**Published:** 2025-01-18

**Authors:** Adrián Cerveró-Varona, Giuseppe Prencipe, Alessia Peserico, Angelo Canciello, Andrew H. House, Hélder A. Santos, Monia Perugini, Ludovica Sulcanese, Chika Takano, Toshio Miki, Annamaria Iannetta, Valentina Russo, Mauro Mattioli, Barbara Barboni

**Affiliations:** 1 Unit of Basic and Applied Sciences, Department of Biosciences and Agro-Food and Environmental Technologies, University of Teramo, 64100 Teramo, Italy; 2 Helsinki University Lipidomics Unit, Helsinki Institute for Life Science (HiLIFE), Biocenter 3, Viikinkaari 1, 00790 Helsinki, Finland; 3 Drug Research Program, Division of Pharmaceutical Chemistry and Technology, Faculty of Pharmacy, University of Helsinki, 00014 Helsinki, Finland; 4 Department of Biomaterials and Biomedical Technology, The Personalized Medicine Research Institute (PRECISION), University Medical Center Groningen (UMCG), University of Groningen, 9713 AV Groningen, the Netherlands; 5 Department of Physiology, Nihon University School of Medicine, Tokyo, Japan; 6 Division of Microbiology, Department of Pathology and Microbiology, Nihon University School of Medicine, Tokyo, Japan; 7 Department of Pediatrics and Child Health, Nihon University School of Medicine, Tokyo, Japan

**Keywords:** Immunology, Cell biology, Functional aspects of cell biology

## Abstract

Amniotic epithelial cells (AECs) exhibit significant immunomodulatory and pro-regenerative properties, largely due to their intrinsic paracrine functions that are currently harnessed through the collection of their secretomes. While there is increasing evidence of the role of bioactive components freely secreted or carried by exosomes, the bioactive cargo of AEC microvesicles (MVs) and their crosstalk with the immune cells remains to be fully explored. We showed that under intrinsic conditions or in response to LPS, AEC-derived MV carries components such as lipid-mediated signaling molecules, ER, and mitochondria. They foster the intra/interspecific mitochondrial transfer into immune cells (PBMCs and Jurkat cells) *in vitro* and *in vivo* on the zebrafish larvae model of injury. The internalization of MV cargoes through macropinocytosis induces hyperpolarization of PBMC mitochondrial membranes and triggers MV-mediated apoptosis. This powerful immune suppressive mechanism triggered by AEC-MV cargo delivery paves the way for controlled and targeted cell-free therapeutic approaches.

## Highlights

• AEC-derived MVs contain functional mitochondria and ER components
• Macropinocytosis drives AEC-derived MVs internalization into immune cells *in vitro*
• AEC-derived MVs suppress immune cell activation via mitochondria-mediated apoptosis
• AEC-derived MVs’ cargo shows immunomodulatory effects *in vivo* in a zebrafish injury model

## Graphical abstract

**Figure:**
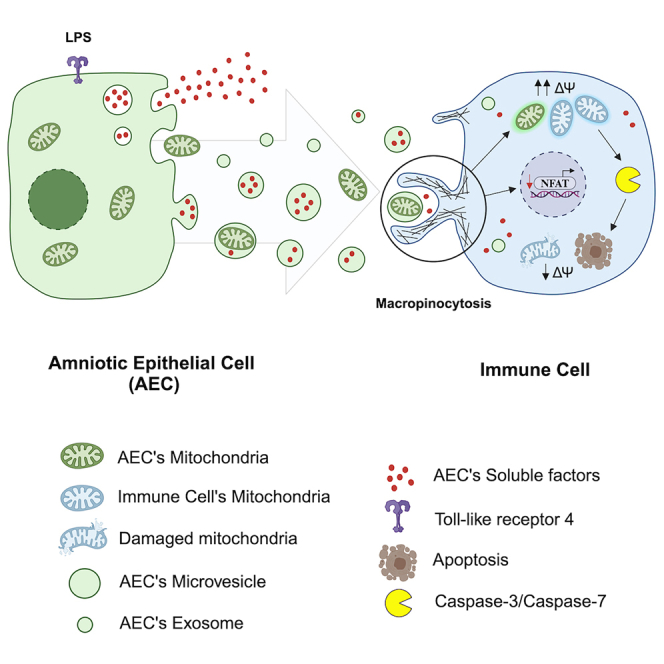

## Introduction

Extracellular vesicles (EVs) are double-membraned nanostructures released from cells serving as vehicles to deliver bioactive contents. They play a crucial role in paracrine intercellular communication in either physiological or pathological conditions.^1^ Based on their composition and biogenesis, EVs are classified into exosomes (Exos, 50–100 nm), microvesicles (MV, 20–1000 nm), and apoptotic bodies (Ab, 1000–5000 nm).^2^ Evidence suggests that EV have a dichotomous regulatory function on immune cells, either enhancing or inhibiting immune responses depending on the cell origin and their cargoes.^3^

In this context, stem cell-derived EV, particularly exosomes, have been confirmed to inhibit the innate immune response (IIR) by carrying immunomodulatory effectors such as growth factors,^4^^,^^5^^,^^6^ transcriptional factors,^7^^,^^8^^,^^9^ mRNA and non-coding RNA,^10^^,^^11^^,^^12^^,^^13^^,^^14^^,^^15^ and cytokines.^16^^,^^17^^,^^18^ Recently, larger EVs such as MV have been recognized for their role in transferring organelles, including mitochondria, lysosomes, endosomal vesicles, endoplasmic reticulum (ER), and plasma membrane as active subcellular components. This transfer efficiently conveys functional cargo from one cell to another generating a powerful intercellular communication mechanism.^19^ MVs released from various cell types, including mesenchymal stem cells (MSC), have been shown to transfer entire active mitochondria, thereby influencing the function of recipient cells.^20^ However, the specific contribution of MV communication to immune responses has rarely been addressed. Only stem cell–derived exosomes have gained attention as potential cell-free tools in regenerative medicine for treating inflammatory diseases, autoimmune disorders, and cancer,^3^^,^^21^ leaving the potential role of MV fraction almost unexplored.

Among the various sources of EV released from mammalian stem cells, perinatal stem cells, including those derived from the placenta and fetal annexes (amniotic and chorionic membranes), have generated growing interest. They have an intrinsic ability to modulate immune response via EV-mediated mechanisms.^3^^,^^22^^,^^23^^,^^24^^,^^25^^,^^26^^,^^27^^,^^28^ This capability is linked to their physiological immunological role essential for the survival of the semi-allogeneic conceptus, the fetus, during pregnancy. Perinatal EV, primarily through their exosomes, have been shown to interact with various innate immune cells, including neutrophils,^29^^,^^30^ peripheral blood mononuclear cells (PBMCs), macrophages, and dendritic cells^24^^,^^26^^,^^28^^,^^31^^,^^32^^,^^33^^,^^34^ either under *in vitro* experiments or *in vivo* preclinical conditions. Their ability to quench the host inflammatory response against pathogens and mechanical insults, and to accelerate/shorten the inflammatory phase and readdress the local immune cells toward a pro-regenerative status,^35^^,^^36^ has sparked interest in their biomedical applicative implications. However, this pro-regenerative immunomodulatory influence that accelerates extracellular matrix remodeling and prepares the framework for tissue homeostasis recovery, has been primarily exploited for EVs obtained from MSC-perinatal derived lineages to date.^37^

Hence, the EV-mediated immunological mechanisms of other perinatal cell types and the role of MV components warrant further exploration. Their large availability and lack of any ethical concerns make them promising candidates for developing innovative cell-free therapeutic protocols.

Based on these premises, the present research aims to characterize the EV released from a specific class of perinatal cells, the epithelial amniotic-derived stem cells (AEC). Their characterization is a prerequisite for investigating the MVs’ role in mediating AEC’s innate immune response and verifying the intracellular mechanisms that transduce the physiological influence of AEC in the control of fetal-maternal immune tolerance. Therefore, to pursue potential MVs-AEC applications, the bioactive influence of MVs’ cargo and the involved immune-modulatory mechanism have been investigated according to stem cell state, by comparing the immune influence of MVs released under native conditions or after LPS-induced inflammatory response in culture.

Here, the immunosuppressive role of AEC-derived MV has been demonstrated, together with macropinocytosis, as large conserved no specific mechanism to internalize them into recipient immune cells (PBMCs and Jurkat cells). The fingerprint of MV cargo is dependent on donor AEC status (intrinsic vs. LPS-induced release) in terms of lipids belonging to signaling pathways, and mitochondrial and ER components. However, MV action in inducing immune cell apoptosis by altering mitochondrial membrane resting potential and hindering NFAT pathway, specific signaling of T cell activation, appears to be steadily conserved in AEC either as intrinsic or LPS-induced condition. Of note, the AEC-derived MV cargo is reproducible *in vivo* on zebrafish larvae where a short mtDNA interspecific heteroplasmy enhances immune cell recruitment to fin-injured sites. Overall, the *in vitro* and *in vivo* results advance knowledge on the immuno-suppressive influence of AEC, a powerful pro-regenerative source of stem cells, establishing a new biological paradigm to develop innovative MV-cell-free therapeutic approaches to face the challenge of immune-mediated disease treatments.

## Results

### MV fraction is a constant component of AEC secretome

**Figure 1:**
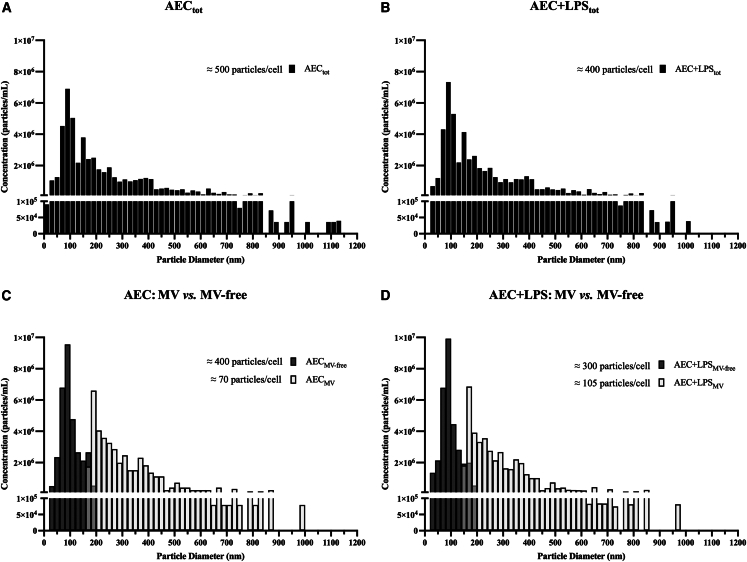
MV fraction is a constant component of AEC secretome (A–D) Exoid was used to characterize the particle size and concentration profiles of (A-B) AEC ± LPS_tot_, (C-D) AEC ± LPS_MV_, and AEC± LPS_MV-free_. a-MEM and PBS were used as background. Data (mean) represent 3 independent sets of experiments (n = at least 3 biological replicates in each group per set; each biological replicate assayed in at least 3 technical replicates).

The distribution of particle peaks in AEC-derived secretomes collected as conditioned media (CM collected from 1.5 x 10^6^ vital AEC ± LPS) (Figure 1A and 1B) showed a size ranging from 30 to about 1000 nm. Notably, many of the particles in the CM fluctuated between 50 and 150 nm, displaying a stable profile independently of cell activation (AEC _tot_ vs. AEC+LPS_tot_; Figures 1A and 1B).

Ultracentrifugation of these CM allowed to purify the MV-enriched fraction (AEC ± LPS_MV_) and the MV-free one (AEC±LPS_MV-free_), as demonstrated by Exoid Tunable Resistive Pulse Sensing (TRPS) characterization (Figures 1C and 1D). Specifically, the MV fractions exhibited particle sizes ranging from 150 to 1000 nm, with a prominent peak around 200 nm. In contrast, the MV-free fraction displayed a particle size distribution ranging from 30 to 200 nm, with a peak around 100 nm (Figures 1C and 1D). Notably, LPS priming caused AEC to secrete a slightly higher number of particles per cell in the MV fraction, while the opposite was observed for the MV-free fraction (Figures 1C and 1D).

### Lipidomic profile of MVs enrichment in lipid mediating signaling and ER/mitochondria is dependent of AEC activation status

**Figure 2:**
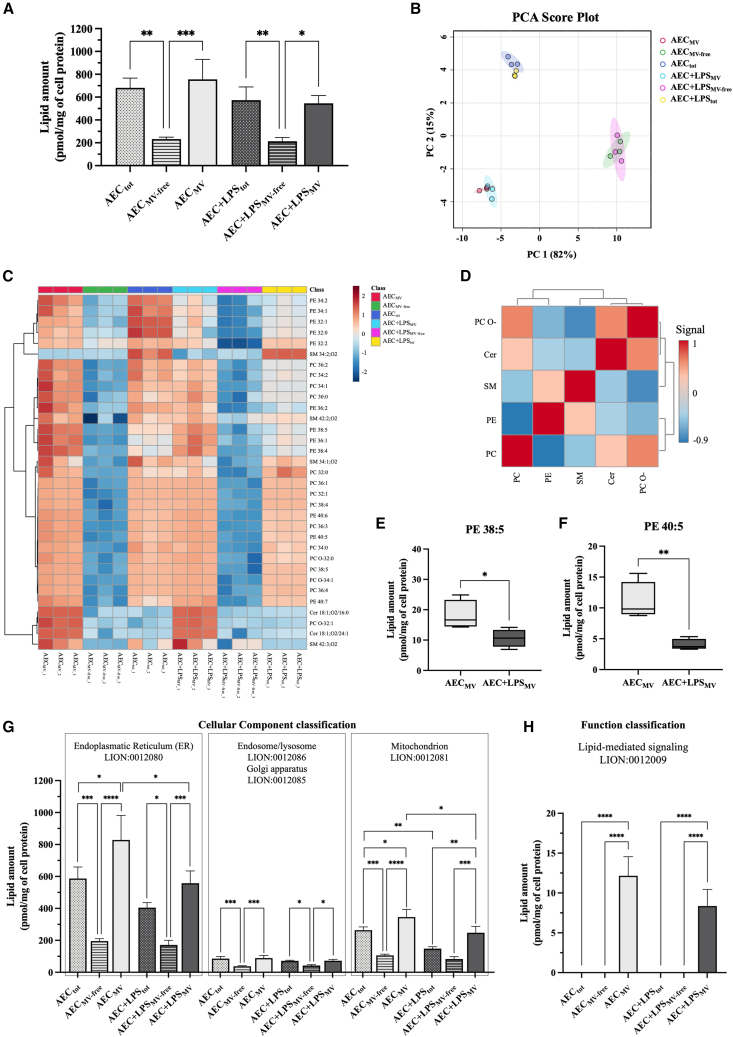
Lipidomic profile of MVs enrichment in lipid mediating signaling and ER/mitochondria is dependent of AEC activation status (A) Total lipid concentration in AEC ± LPS_tot_, AEC± LPS_MV-free,_ and AEC ± LPS_MV_ fractions. (B) Bidimensional principal component analysis (PCA) of all fractions. (C) Hierarchical heatmap with Pearson’s coefficient of the lipidic fingerprint for each specific lipid in all fractions. (D) Spearman’s rank correlation analysis of the different lipid classes influenced by all CM fractions. (E and F) Lipid concentration for PE 38:5 and PE 40:5, respectively, in the AEC ± LPS_MV_ fractions. (G) Cellular component classification AEC_tot_, AEC_MV- free,_ and AEC_MV_ fractions treated ± LPS assessed with the Lipid Ontology web (LION/web) terms. (H) Function classification for the “lipid-mediated signaling” term of the lipid ontology web (LION/web). Data (mean ± SD) represent 3 independent sets of experiments (n = at least 3 biological replicates in each group per set; each biological replicate assayed in at least 3 technical replicates). ∗, ∗∗, ∗∗∗, and ∗∗∗∗ Statistically significant values between the different studied groups (*p* < 0.05, *p* < 0.01, *p* < 0.001, and *p* < 0.0001, respectively). One-way ANOVA and two tailored t-tests were employed for comparing normally distributed data.

Lipidomic analysis of whole AEC-derived secretome (AEC_tot_) showed that the overall lipid concentration (pmol/mg of cell protein) was dependent on the different fractions and the inflammatory cell response (Figure 2A). In detail, the MV-free fractions (AEC_MV-free_) contained lipid levels significantly lower than CM (AEC_tot_) and MV fractions (*p* < 0.01 vs*.* AEC_tot_ and *p* < 0.001 vs*.* AEC_MV_). Moreover, the assessment of the lipid heterogenicity in the secretome fractions was further investigated by adopting a dimensionality reduction Principal Component Analysis (PCA) (Figure 2B) and a hierarchical heatmap (Figure 2C). In detail, PCA analysis showed that 96% of the total variation was obtained using only two principal components (Figure 2B) which generated three distinct clusters corresponding to each fraction (AEC ± LPS_tot_, AEC±LPS_MV-free_, and AEC ± LPS_MV_), indicating the substantial differences in lipid composition between those fractions. Similarly, the hierarchical heatmap showed that the MV-free fractions (AEC±LPS_MV-free_) exhibited a lower overall concentration in all lipid species, whereas the AEC ± LPS_MV_ fraction resulted to be enriched in Cer d18:1/16:0, Cer d18:1/24:1, PC O-32:1, and SM 42:3 (Figure 2C). Moreover, the comparative analysis confirmed that LPS activation modified the organelle-derived cargo (Figure 2C). Indeed, AEC vs. AEC+LPS groups revealed differential upregulation in lipids belonging to PE 34:2, PE 34:1, PE 32:1, and PE 32:0. Of note, the correlation heatmap, summarized in Figure 2D, also revealed that some lipid classes were correlated with each other’s, such as PC and PE (inverse correlation) and PC and PC-O (positive correlation). Notably, polyunsaturated fatty acid (PUFA) chains and substrates for arachidonic acid (AA) were significantly less represented in AEC+LPS_MV_ (*p* < 0.05 and *p* < 0.01 vs. AEC_MV_, respectively) as indicated from PE 38:5 and PE 40:5 concentrations (Figures 2E and 2F).

Transitioning to the computational biology analysis by using the LION/web database (http://www.lipidontology.com/), the subcellular origin of detected lipids was elucidated (Figure 2G). The results showed that lipids belonging to the ER (LION:0012080) were the most represented, as demonstrated by the prevalence of PC and PE classes accounting for approximately 50% of the total lipid levels. They were mainly recorded in the MV fraction of AEC with a significant reduction after LPS treatment (*p* < 0.05, Figure 2G). Mitochondria-derived lipids (LION:0012081) were the second biological class of recorded lipids, represented by the PE class accounting for around 25% of the total composition. Analogously, they were significantly higher in AEC_MV_ than in LPS-activated AEC_MV_ (*p* < 0.05, Figure 2G).

Finally, the biological function of lipid composition was interpreted using TERMS from the LION/web database. Notably, the “lipid-mediated signaling” (LION:0012009) was exclusively recorded in the MV fractions with higher values in AEC_MV_ (*p* < 0.0001 vs. both AEC_tot_, AEC_MV-free,_ Figure 2H).

### Protein content-based MV fraction characterization confirmed the presence of functional mitochondria

**Figure 3:**
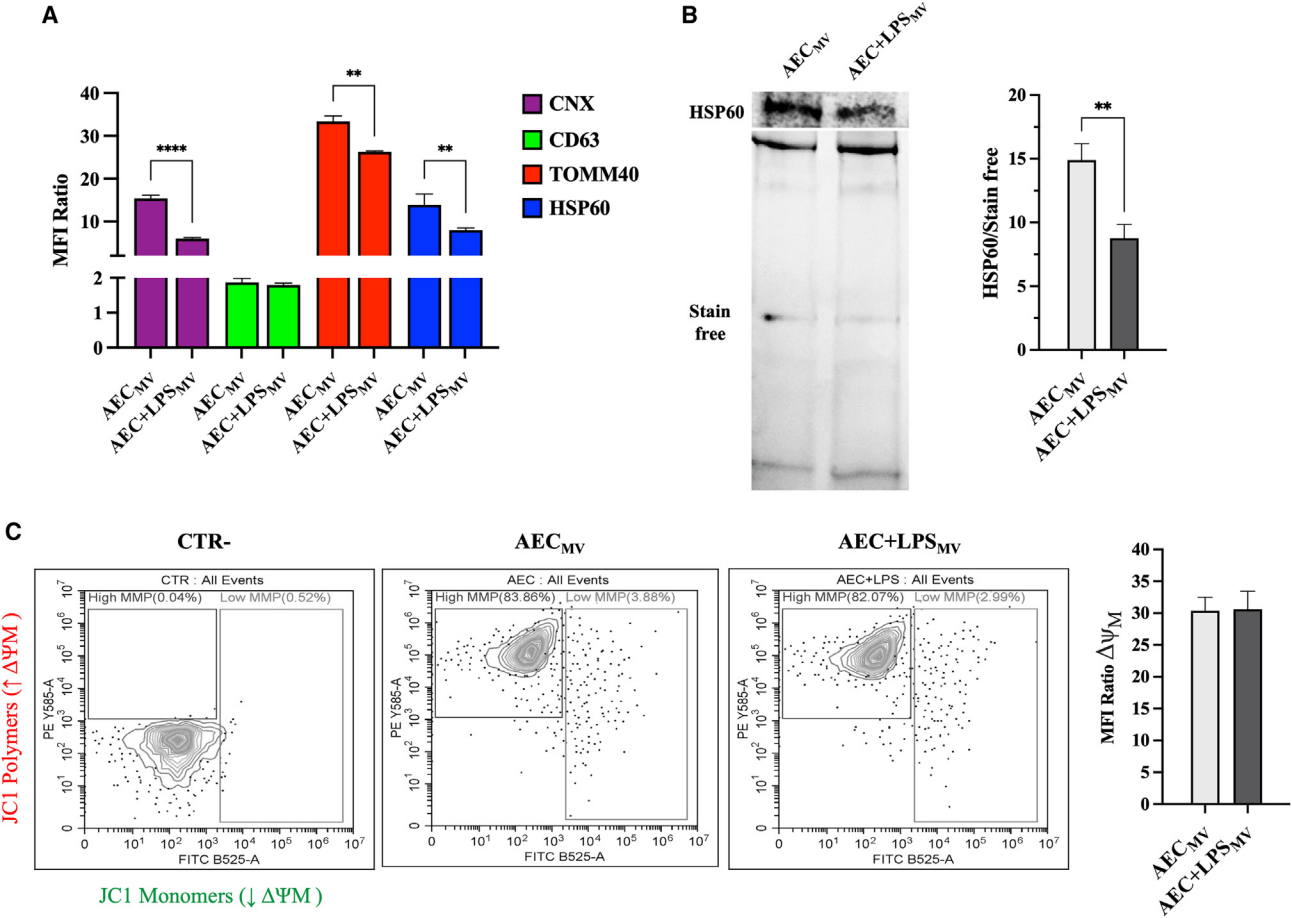
Protein content-based MV fraction characterization confirmed the presence of functional mitochondria (A) Flow cytometric analysis of CNX, CD63, TOMM40, and HSP60, in AEC ± LPS_MV_. Data were presented as mean fluorescence intensity (MFI) ratio over negative CTR. (B) Representative WB images and relative densitometric analysis of HSP60 (normalized on total protein content via stain-free detection) in AEC ± LPS_MV_. (C) JC1 flow cytometric analysis to evaluate AEC ± LPS_MV_ ΔΨM. Data were presented as MFI ratio over negative CTR. Data (mean ± SD) represent 3 independent sets of experiments (n = at least 3 biological replicates in each group per set; each biological replicate assayed in at least 3 technical replicates). ∗∗ and ∗∗∗∗ Statistically significant values between the different studied groups (*p* < 0.01 and *p* < 0.0001). One-way ANOVA and two tailored t-tests were employed for comparing normally distributed data.

Following the lipidomic prediction, we conducted a comprehensive protein composition analysis of the MV fraction to confirm the presence of ER, EVs, and mitochondrial components. As shown in Figure 3A, flow cytometric analysis demonstrated the presence of proteins associated with the ER (CNX), the membrane envelope (CD63: a marker of intracellular vesicle membranes), and mitochondria (TOMM40 and HSP60). Notably, the analysis of mitochondrial protein markers carried out with either a cytofluorimeter or western blotting confirmed their higher presence in basal MV fraction (*p* < 0.01 vs. AEC+LPSMV; Figures 3A and 3B).

Moreover, JC1 flow cytometric analysis was performed to assess the functionality of the mitochondria within the MV fractions, it revealed a high mitochondrial membrane potential (ΔΨM) in both AEC_MV_ and AEC+LPS_MV_ fractions (Figure 3C). This demonstrated the presence of functional mitochondria within the MVs, with no significant differences observed between AEC and AEC+LPS, suggesting that LPS treatment affected mitochondrial concentration, but not its function.

### Mitochondrial tracking demonstrated the AEC-derived MV cargo internalization in immune cells

Given the evidence that MV derived from native or activated AEC carried functional components, the study moved toward the investigation of their cargo trafficking into recipient immune cells (PBMCs and Jurkat) using the enclosed mitochondria for tracking.

**Figure 4:**
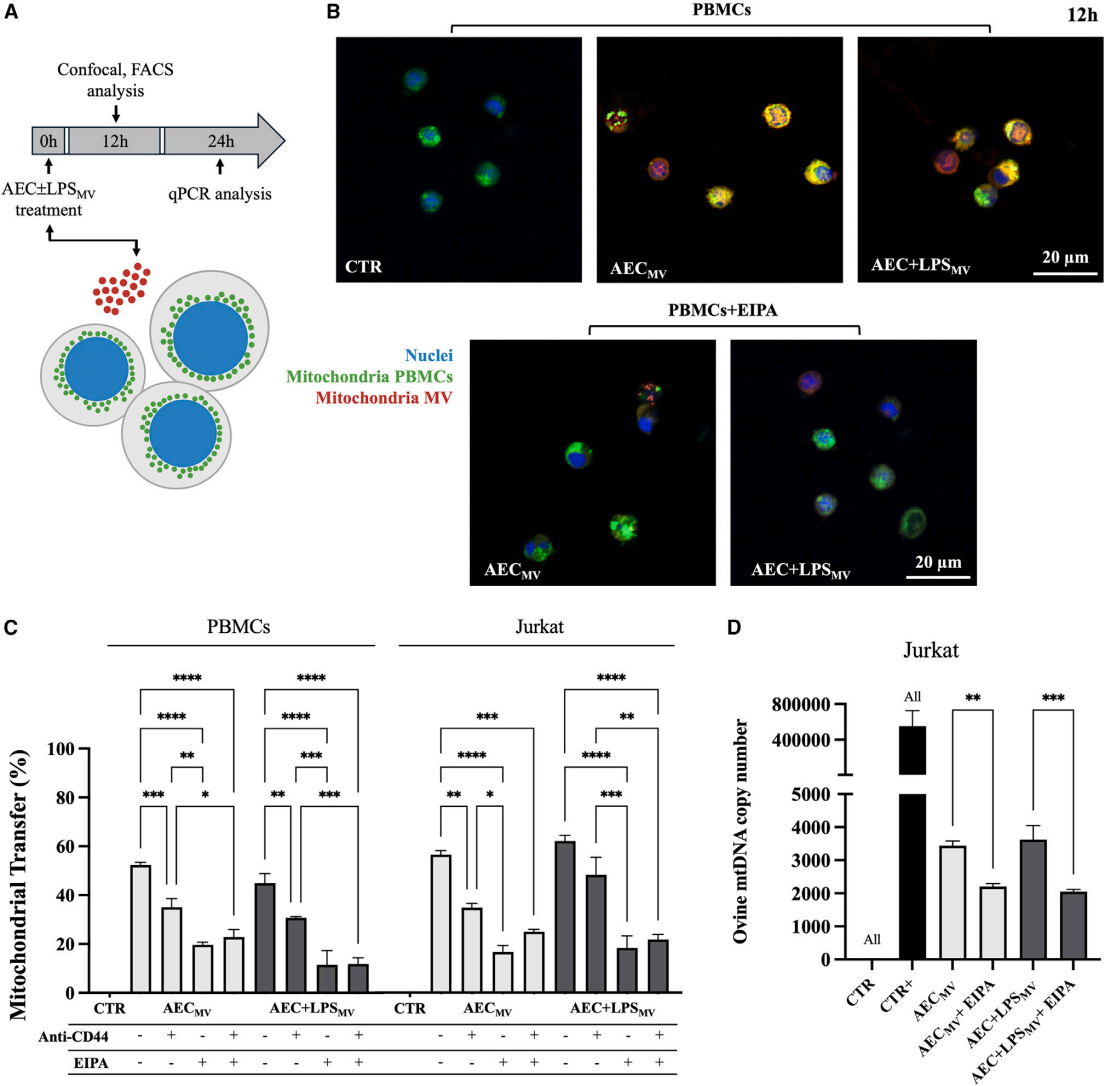
Mitochondrial tracking demonstrated the AEC-derived MV cargo internalization in immune cells (A) Experimental design for B-D. (B) Representative confocal images of co-immunofluorescence staining of nuclei (DAPI), MitoTracker green (PBMCs’ mitochondria), and MitoTracker red (MV’ mitochondria) in PBMCs ± EIPA exposed to AEC ± LPS_MV_ for 12h. (C) Flow cytometric investigation to confirm the mitochondria MV internalization (MitoTracker red) ± LPS in PBMCs and Jurkat ± CD44 and ± EIPA. Data are presented as % of cells positive to MitoTracker red. (D) qPCR analysis to corroborate the mitochondria MV internalization after 24h of exposition to AEC ± LPS_MV_ by examining the ovine mtDNA copy number in Jurkat ± EIPA. (CTR: untreated Jurkat; CTR+: AEC_MV_ per se). Data (mean ± SD) represent 3 independent sets of experiments (n = at least 3 biological replicates in each group per set; each biological replicate assayed in at least 3 technical replicates). ALL, ∗, ∗∗, ∗∗∗, and ∗∗∗∗ Statistically significant values between the different studied groups (*p* < 0.0001, *p* < 0.05, *p* < 0.01, *p* < 0.001, and *p* < 0.0001, respectively). For the transfer of AEC_MV_ mitochondria into AEC see Figure S1. One-way ANOVA was employed for comparing normally distributed data. Scale bar, 20 μm.

Indeed, the uptake of MV-derived cargo in immune cells was investigated by analyzing the fluorescence of MV pre-labeled mitochondria (red MitoTracker) in PBMCs (Figure 4B) or, in addition, by demonstrating the interspecific heteroplasmy (ovine mtDNA copy numbers) in recipient Jurkat cells (Figure 4D).

Confocal analyses (Figure 4B) revealed the internalization of MV-derived mitochondria (red) into PBMCs after 12 h of co-incubation, which strongly co-localized with the endogenous ones (green; orange as merge fluorescence). Of note, the specificity of MV-mediated mitochondria transfer was confirmed using the EIPA macropinocytosis inhibitor, which massively prevented the fluorescence uptake.

Flow cytometer quantitative analysis was carried out to detect the uptake of red MitoTracker in immune recipient cells (see also Figure S1 for mitochondria uptake into AEC). The analysis confirmed the mitochondria intra and interspecific trafficking between AEC ± LPS_MV_ and PBMCs (ovine vs. ovine: AEC ± LPS_MV_ vs. PBMCs), or Jurkat (ovine vs. human: AEC ± LPS_MV_ vs. Jurkat). This uptake was not influenced by the functional state of the AEC that released the MV. No significant difference was observed between the internalization of AEC_MV_ and AEC+LPS_MV_ mitochondria (*p* > 0.05; Figure 4C). The widespread internalization of mitochondria achieved with immune cells exposure to AEC ± LPS_MV_ fraction had been inhibited more efficiently with EIPA than with CD44 treatment (*p* < 0.05; Figure 4C), thus emphasizing the prominent role of macropinocytosis in this intercellular dialogue.

The interspecific transfer of ovine mtDNA was quantified using the species-specific copy numbers, thus providing solid evidence of mitochondrial internalization^38^ in Jurkat cells (Figure 4D). Analogously, the quantification of transferred ovine mtDNA copy numbers confirmed that this intercellular paracrine communication is not affected by the functional status of AEC (native AEC vs. LPS-activated ones). Indeed, transcellular transfer of mitochondria induced by MV fractions derived from both AEC and AEC+LPS in 24 h involved the same amount of ovine mtDNA.

**Figure 5:**
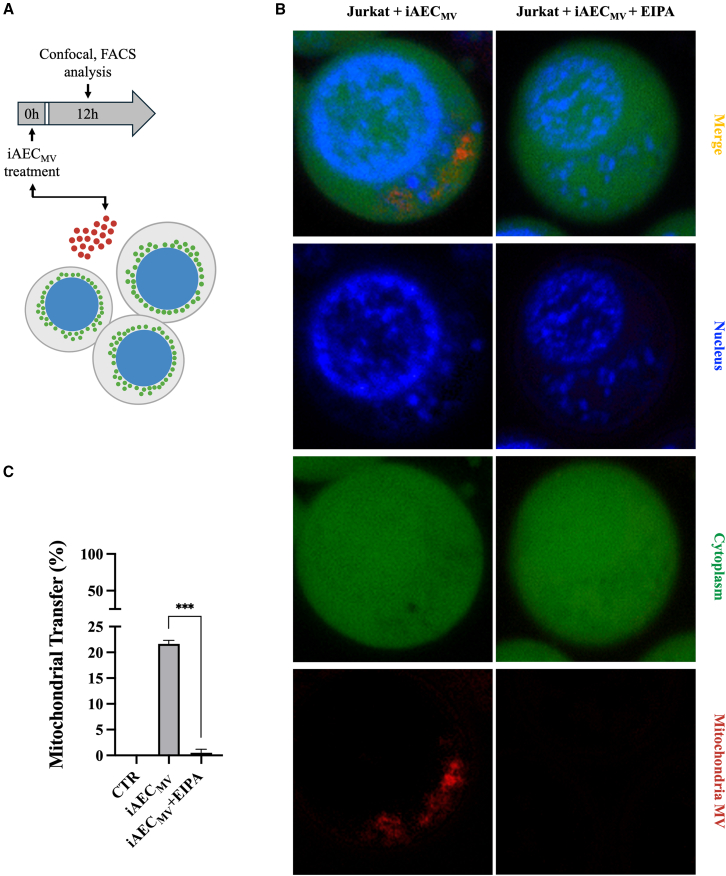
Mitochondrial tracking demonstrated the iAEC-derived MV cargo internalization in immune cells (A) Experimental design for B-C. (B) Representative confocal images of co-immunofluorescence staining of nuclei (Hoescht 33342), cytoplasm (Calcein AM), and mitochondria-targeted turbo red florescence protein (TurboRFP-labeled mitochondria), in Jurkat ± EIPA exposed to iAEC_MV_ for 12h. (C) Flow cytometric investigation to confirm the mitochondria MV internalization (TurboRFP-labeled mitochondria) in Jurkat ± EIPA. Data are presented as % of cells positive to TurboRFP. Data (mean ± SD) represent 3 independent sets of experiments (n = at least 3 biological replicates in each group per set; each biological replicate assayed in at least 3 technical replicates). ∗∗∗ Statistically significant values between the different studied groups (*p* < 0.001). One-way ANOVA was employed for comparing normally distributed data. Scale bar, 5 μm.

Finally, to genetically validate the intercellular transfer of viable mitochondria (Figure 5A), we employed an immortalized human AEC (iAEC) line expressing mitochondria-targeted turbo red fluorescent protein (turboRFP), kindly donated by the Toshio Miki Lab at Nihon University School of Medicine, Tokyo, Japan. Confocal microscopy (Figure 5B) and FACS analyses (Figure 5C) demonstrated that the turboRFP-labeled mitochondria were successfully transferred to Jurkat cells, confirming the occurrence of intercellular mitochondrial transfer. Of note, EIPA completely blocked the uptake of labeled mitochondria, further confirming the involvement of macropinocytosis (*p* < 0.001; Figures 5B and 5C).

### Interspecific *in vivo* transfer of AEC-MV mitochondria cargo

**Figure 6:**
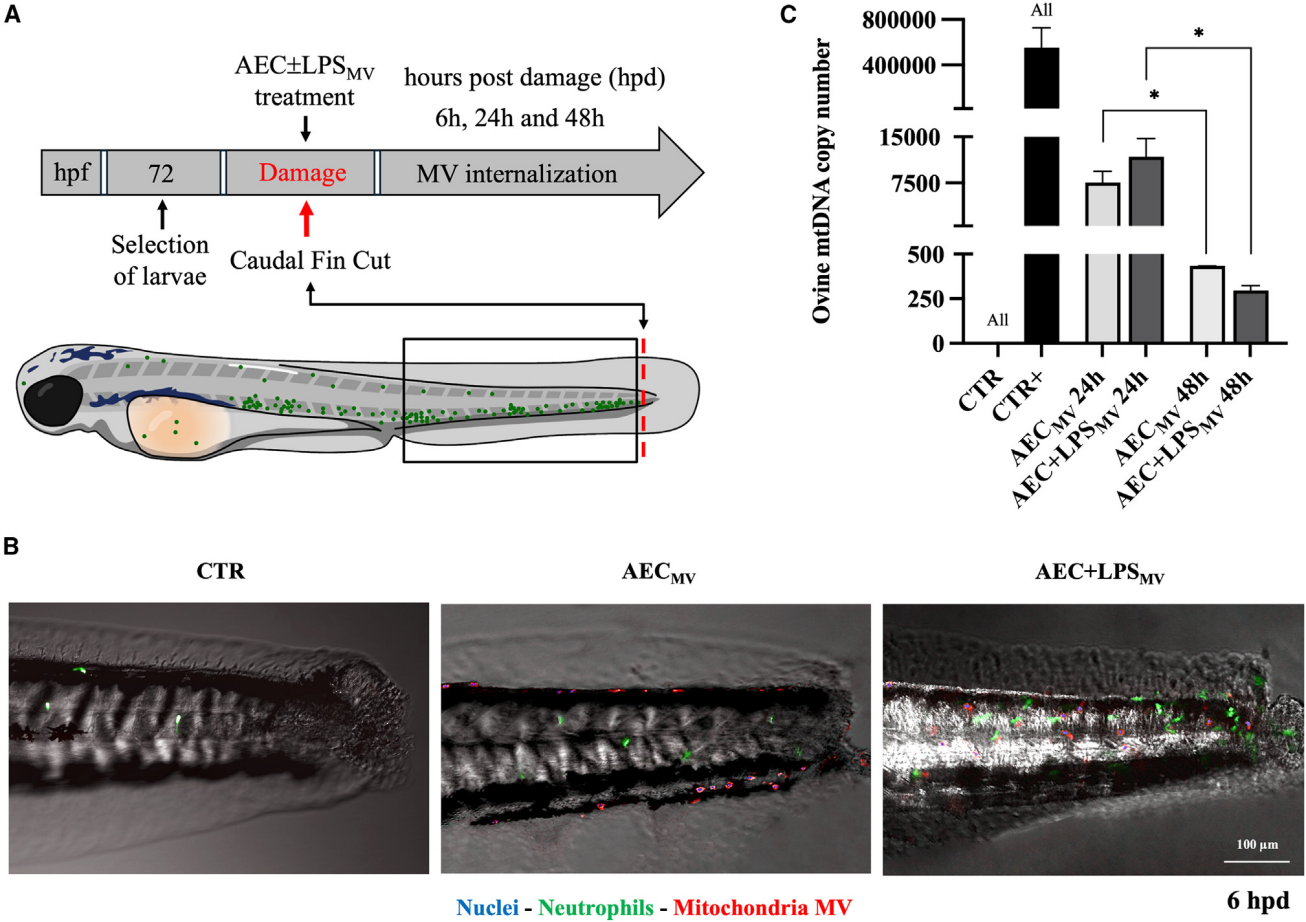
Interspecific in vivo transfer of AEC-MV mitochondria cargo (A) Illustration describing the experimental plan followed for the treatment of 72 hpf zebrafish larvae with AEC ± LPS_MV_, and time points for the subsequent analyses. (B) Representative confocal microscopy images of Tg (mpx:GFP) larvae after 6 h post damage (caudal fin cut) and treatment with AEC ± LPS_MV_ reportig the localization of ovine mitochondria MV (MitoTracker red), zebrafish neutrophils (green) and cell nuclei (blue) in the caudal fin. (C) Quantification by qPCR of the amount of ovine mtDNA copy number contained in the zebrafish larvae after 24 and 48 hpd. (CTR: untreated zebrafish larvae; CTR+: AEC_MV_ per se). Data (mean ± SD) represent 3 independent sets of experiments (n = at least 10 biological replicates in each group per set). All, and ∗ Statistically significant values between the different studied groups (*p* < 0.0001, and *p* < 0.05, respectively). One-way ANOVA was employed for comparing normally distributed data. Scale bar, 100 μm.

The working mechanism of transcellular transfer of mitochondrial cargo derived from AEC ± LPS_MV_ was finally investigated *in vivo* using a zebrafish animal model under acute inflammation. The transgenic line *Tg(mpx:GFP)* which enables tracking of neutrophils that express green fluorescent protein (GFP) under the neutrophil-specific myeloperoxidase (mpx) promoter, was chosen to follow mitochondria distribution and provide an internal reference for anatomical orientation and blood vessel district identification. The caudal fin of zebrafish larvae was cut at 72 hours postfertilization (hpf) to mechanically reproduce an acute inflammatory response. Then, mitochondrial internalization was detected by pre-labeling AEC ± LPS_MV_ mitochondrial cargoes with red MitoTracker (Figures 6A and 6B) or by detecting the presence of specie-specific mtDNA (ovine mtDNA). The qualitative analysis of early mitochondrial transfer after MV exposure was carried out using a confocal microscope at 6 h post damage (hpd) (Figure 6B). Internalized mitochondria were mainly recorded in the wounded caudal fin area. However, migrating mitochondria were also located inside round structures (10–15 μm), blood circulatory cells, enriched in red fluorescence with a central nucleus counter-stainable with DAPI (Figure 6B).

To quantify the ovine mitochondria transferring after exposure to MVs derived from different cell donors (AEC ± LPS) and, especially, to determine their long-term retention, qPCR was performed at 24 and 48 hpd^38^ by leveraging interspecific chimerism (ovine mtDNA into zebrafish). The successful transfer of ovine mitochondria (*p* < 0.0001; Figure 6C) was detected at 24 hpd with the internalization of mtDNA copies independent of the functional status of MV-donor AEC (AEC_MV_ or AEC+LPS_MV_). Ovine mtDNA significantly decreased passing from 7000 to 1000 at 24 hpd to 300–400 copy numbers in the following 24 h (48 hpd), demonstrating the non-integration and prompt degradation of heteroplasmic mitochondria (*p* < 0.05, Figure 6C) in both experimental groups (AEC_MV_ and AEC+LPS_MV_).

Notably, the use of EIPA in zebrafish larvae exposed to MVs did not demonstrate efficacy at doses up to 100 μM, even after 30 h of exposure (data not shown). This suggests that, within the tested parameters, EIPA does not exert its therapeutic effect in this *in vivo* experimental setup.

### AEC_MV_ cargo transfer promoted an immunosuppressive response in PBMCs and Jurkat cells

**Figure 7:**
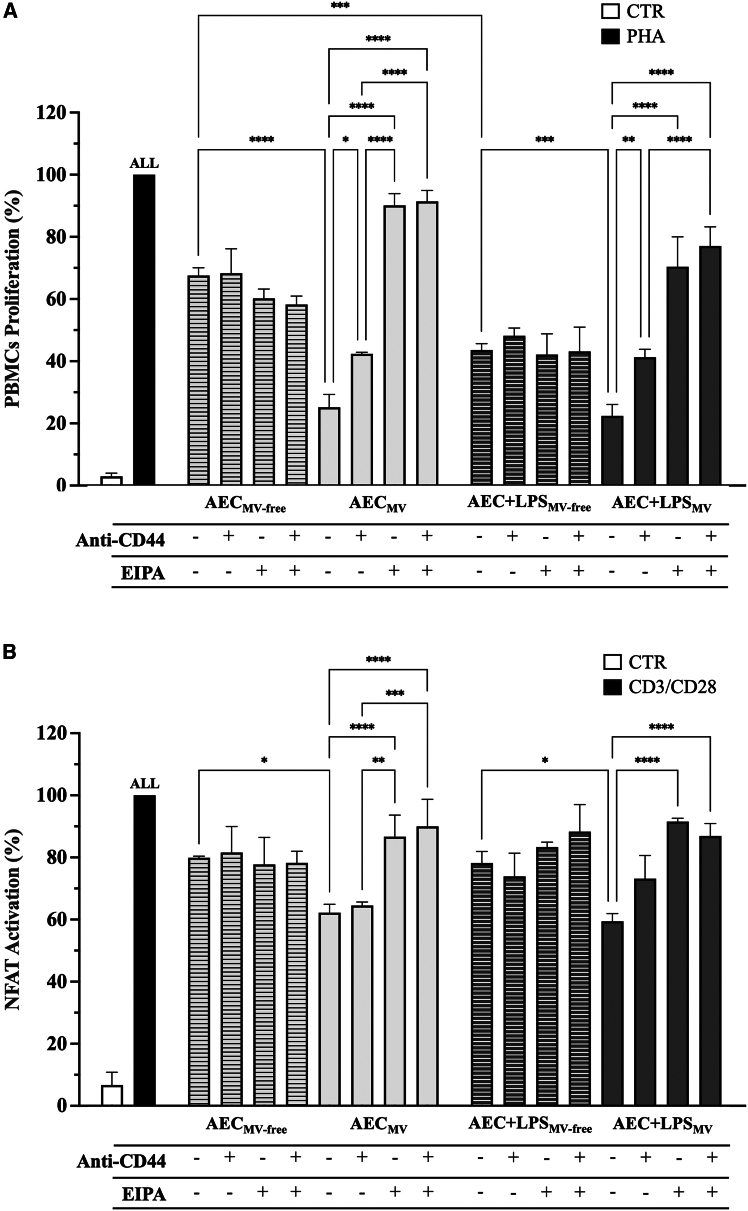
AEC_MV_ cargo transfer promoted an immunosuppressive response in PBMCs and Jurkat cells (A) PHA-stimulated PBMCs ± Anti-CD44 and ± EIPA were treated with the different CM (AEC ± LPS_MV_ and AEC± LPS_MV-free_) for 48h and assessed for their proliferation. Data were normalized on PHA-stimulated PBMCs (100% of proliferation). (B) CD3/CD28-stimulated Jurkat reporter cells ± Anti-CD44 and ± EIPA were evaluated for the inhibition of NFAT activation after treatment with the different CM (±LPS) for 48h. Data were normalized on CD3/CD28 stimulated Jurkat (100% of NFAT activation). Data (mean ± SD) represent 3 independent sets of experiments (n = at least 3 biological replicates in each group per set; each biological replicate assayed in at least 3 technical replicates). ALL, ∗, ∗∗, ∗∗∗, and ∗∗∗∗ Statistically significant values between the different studied groups (*p* < 0.01, *p* < 0.05, *p* < 0.01, *p* < 0.001, and *p* < 0.0001, respectively). One-way ANOVA was employed for comparing normally distributed data.

Given the confirmation of AEC_MV_ internalization, the study proceeded to investigate the immunomodulatory influence of this intercellular communication mechanism by comparing the impact of MV and MV-free fractions on activated intra and interspecific immune cells (ovine PBMCs and human Jurkat). As illustrated in Figure 7A, although the MV-free fraction was able to significantly prevent the proliferation of PBMCs triggered by PHA, this inhibitory influence never exceeded 50%. Whereas the MVs’ fraction displayed a strong inhibitory action which was significantly more potent ranging between 70 and 80% (AEC_MV-free_ vs. AEC_MV_: *p* < 0.0001, and AEC+LPS _MV-free_ vs. AEC+LPS _MV_: *p* < 0.001). Of note, the inhibitory influence exerted by the MV-free fraction was strictly dependent on the status of AEC (AEC_MV-free_ vs. AEC+LPS_MV-free_: *p* < 0.001), whereas the MV one exerted a constant massive inhibitory influence without any difference upon the origin of donor cells (AEC_MV_ vs*.* AEC+LPS _MV_: *p* > 0.05; Figure 7A). This result indicates that AEC exposed to pro-inflammatory LPS stimulus adapt its immunomodulatory function by acting on the bioactive components freely secreted or conveyed by exosomes rather than by modifying the cargo MV elements.

The AEC-derived MVs exert the immunosuppressive role once internalized in immune recipient cells. Indeed, the use of pinocytosis inhibitors (anti-CD44 and EIPA) significantly reduced their immunomodulatory effect (AEC ± LPS_MV_ vs. + Anti-CD44: *p* < 0.05, and AEC ± LPS_MV_ vs. + EIPA: *p* < 0.0001; Figure 7A). Of note, EIPA was demonstrated to be the most powerful inhibitor by almost totally removing the immunosuppressive influence of MVs (*p* < 0.0001 vs. CD44; Figure 7A), underpinning macropinocytosis as the primary mechanism for intercellular MV cargo trafficking.

Finally, the use of interspecific Jurkat reporter cells was tempted to examine the degree of conservation of MV-mediated immune suppressive mechanism. In addition, this experimental setup was carried out to verify whether the transfer of MV cargo interferes with specific intracellular immune signaling pathways, such as the nuclear factor of activated T cells (NFAT) once activated by CD3/CD28.^39^^,^^40^^,^^41^^,^^42^ In detail, both the MV-free and MV fractions were able to inhibit NFAT activation (Figure 7B), although the MV one exhibited a significantly higher effect (*p* < 0.05; Figure 7B). This effect can be hindered by preventing macropinocytosis with EIPA (*p* < 0.0001; Figure 7B).

These findings reveal that the immunosuppressive role of AEC is exerted using bioactive components released either in the MV-free fraction (soluble factors and exosomes) or the MV fraction. MV enables intercellular crosstalk with immune cells by strongly preventing their activation. Particularly, MVs inhibit the proliferative response of PBMCs and Jurkat cells induced by PHA or CD3/CD28 stimuli, respectively, by interfering with NFAT intracellular signaling pathways in the latter cells. This immune suppression is achieved after the transcellular transfer of MV cargoes via macropinocytosis. Although the MV cargo selection in response to inflammatory AEC activation have been demonstrated, the immune-suppressive role mediated by MVs is independent from AEC donor cells (±LPS). On the contrary, MV fraction of AEC secretomes maintains a stable immune suppressive influence exerting a consistent inhibitory feedback loop controlling immune cells.

### Exposure of injured zebrafish larvae to AEC-derived MV stimulated early immune cell recruitment

**Figure 8:**
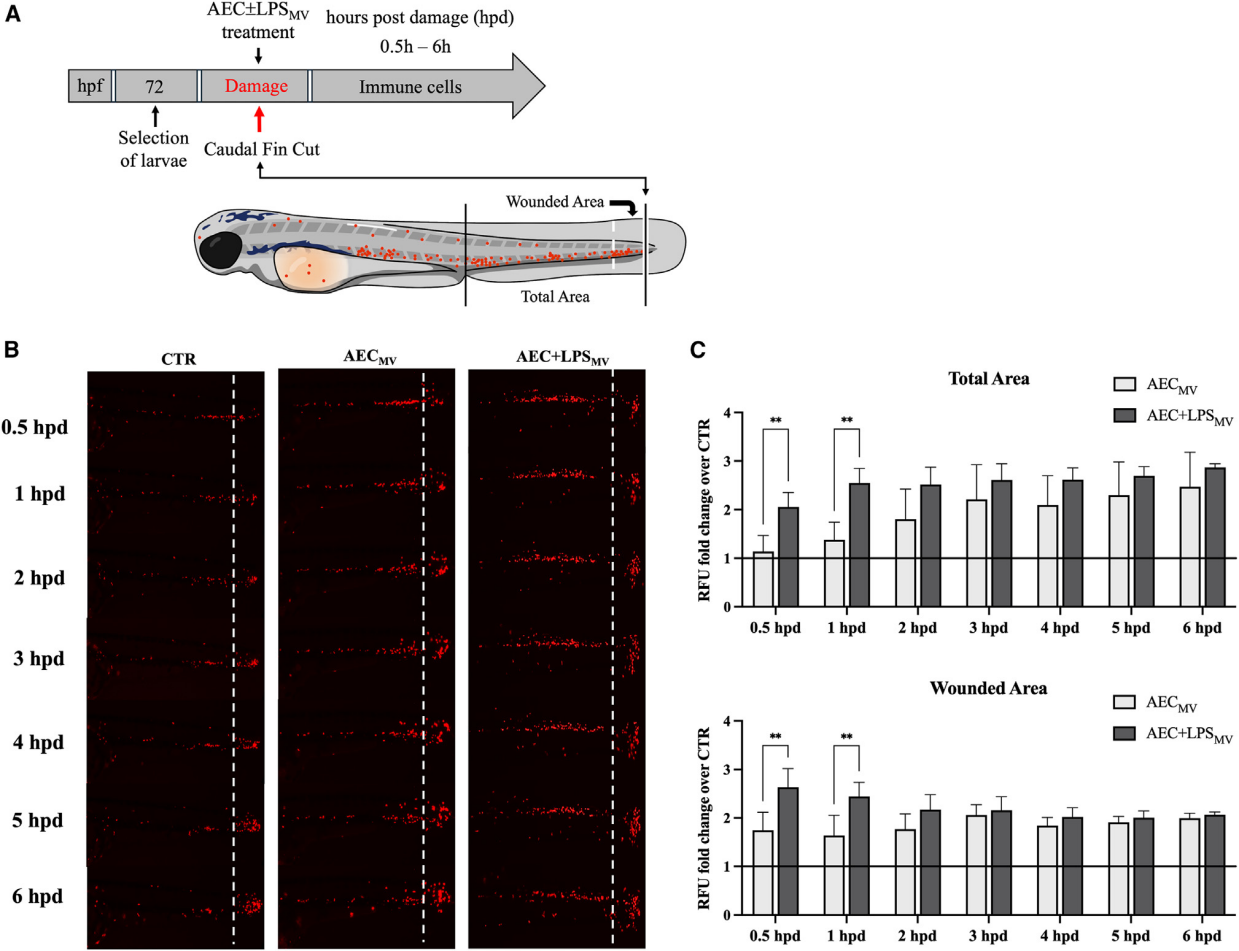
Exposure of injured zebrafish larvae to AEC-derived MV stimulated early immune cell recruitment (A) Illustration depicting the experimental plan followed for the treatment of 72 hpf zebrafish larvae with AEC ± LPS_MV_ and subsequent time lapse observation. (B) Exemplary images of *Tg(lysz:dsRed2*) larvae post tail fin resection, showcasing the localization of immune cells in the tail and their accumulation at the site of injury from 0.5 to 6 hpd. (C) Densitometric analysis of RFU, representing the amounts of immune cells detected in the total area and within the wounded area, normalized over CTR (black line). Data (mean ± SD) represent 3 independent sets of experiments (n = at least 3 biological replicates in each group per set). ∗, ∗∗ Statistically significant values between the different studied groups (*p* < 0.05, *p* < 0.01, respectively). One-way ANOVA was employed for comparing normally distributed data. Scale bar, 100 μm.

The immune-suppressive influence of MVs was further assessed using the *in vivo* setting of acute inflammation. To this aim, the pan-leukocytic transgenic zebrafish larvae line *Tg(lyz:dsRed2*) that express fluorescent dsRed2 protein under the lysozyme C promoter, was employed as model to quantify the early immune cells recruitment in response to caudal fin cut (Figure 8A). The analyses were carried out also by verifying that ovine mtDNA copies transfer occurred, as previously demonstrated (Figure 6C), independently of the cell donors’ status (data not shown).

Of note, the zebrafish larvae exposed to AEC ± LPS_MV_ displayed an accelerated immune cells recruitment from 0.5 hpd to 6 hpd (Figures 8B and 8C). In particular, the relative fluorescence intensity (RFU) levels detected in both caudal total and wounded area at 0.5 hpd and 1 hpd demonstrated the highest migratory activity of immune cells in larvae exposed to AEC+LPS_MV_ (*p* < 0.01 vs. AEC_MV_ and *p* < 0.0001 vs. CTR; Figure 8C). The analyses performed subsequently showed that the MV-exposed larvae maintained a significant higher infiltration of immune cells but, in this phase, totally independent of the status of MV donor AEC (Figure 8C).

### Induction of apoptosis in PBMCs via MV internalization

**Figure 9:**
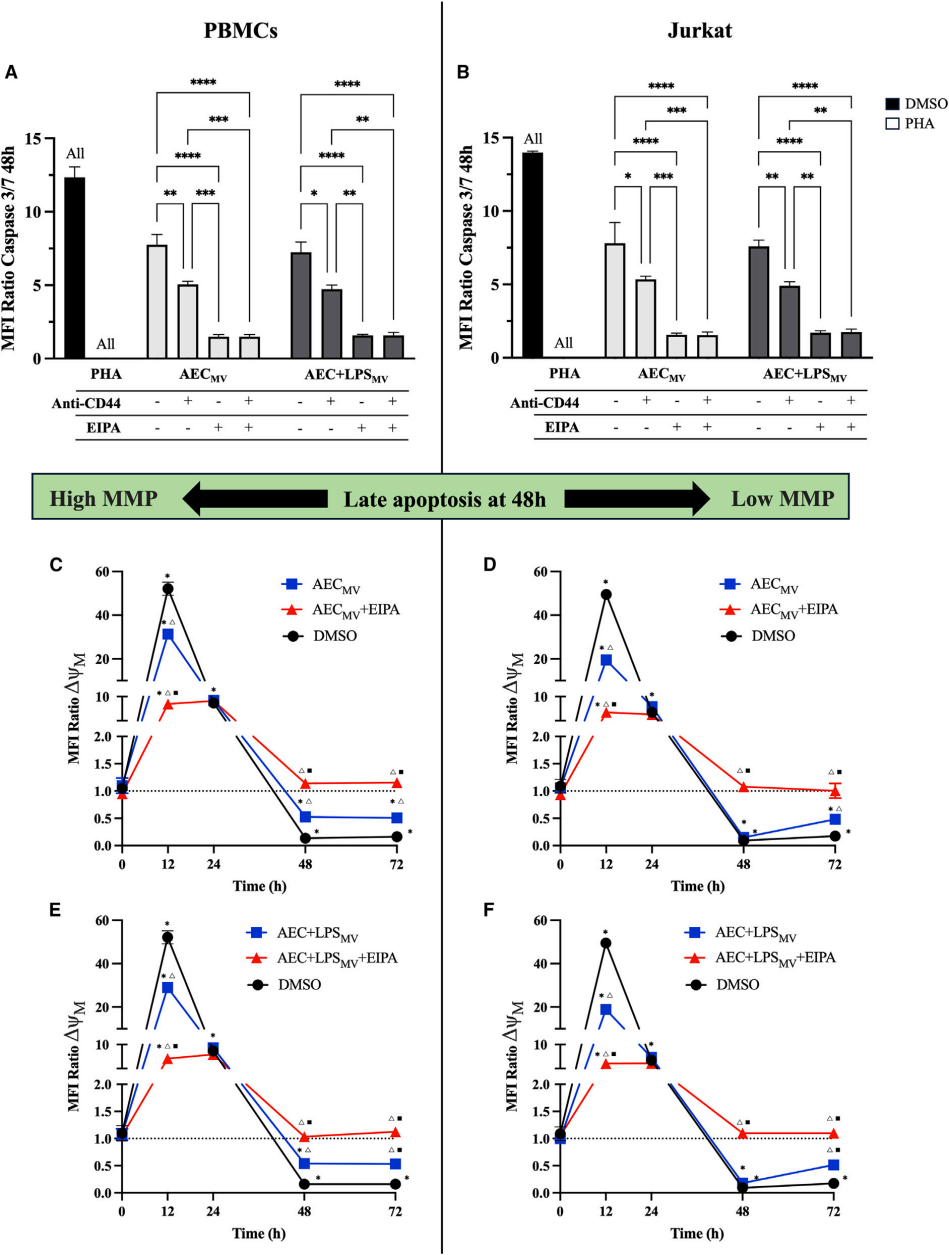
Induction of apoptosis in PBMCs via MV internalization (A and B) Flow cytometric analysis of Caspase 3/7 in PBMCs and Jurkat ± Anti-CD44 and ± EIPA exposed to AEC ± LPS_MV_ for 48h. Data are presented as MFI ratio over negative CTR. (C–E and D–F) JC1 flow cytometric analysis to evaluate the ΔΨM of PBMCs and Jurkat ± EIPA exposed to AEC ± LPS_MV_ for 12-72h. Data were presented as MFI ratio over negative CTR. Values were normalized on the basal ΔΨM of activated PBMCs. Data (mean ± SD) represent 3 independent sets of experiments (n = at least 3 biological replicates in each group per set; each biological replicate assayed in at least 3 technical replicates). (A) ALL, ∗, ∗∗, ∗∗∗, and ∗∗∗∗ Statistically significant values between the different studied groups (*p* < 0.0001, *p* < 0.05, *p* < 0.01, *p* < 0.001, and *p* < 0.0001, respectively). (B) ∗, ^Δ^, and ^▪^*p* < 0.05 between the groups studied (PHA, DMSO, AEC ± LPS_MV_, respectively). No apoptotic effect was recorded following AEC_MV_ mitochondria transfer into AEC (see Figure S1). One-way ANOVA was employed for comparing normally distributed data.

Once the AEC-mediated immunomodulatory influence by the internalization of MV cargo was ascertained, the study moved toward the immune cell mechanism involved. In particular, the role of apoptosis in mediating the inhibitory effect of AEC ± LPS_MV_ was investigated on PBMCs and Jurkat. The caspase 3/7 activation detected via flow cytometer, demonstrated that the exposure of activated immune cells to MV fractions (±LPS) promoted in 48 h a high incidence of late apoptosis that reached about half levels than that achieved by exposing the cells to high concentrations of DMSO (Figures 9A and 9B). The induction of apoptosis recorded after MV exposure to PBMCs and Jurkat was almost totally prevented (more than 80%) when the macropinocytosis of their cargo was impaired using EIPA (*p* < 0.0001; Figures 9A and 9B), highlighting the strong association between MV cargo internalization and the induction of immune cells’ programmed death. Of note, no apoptosis was recorded following MV transfer into AEC.

The intracellular mechanisms underlying the induction of apoptosis have been further investigated by recording the early time course of mitochondrial membrane potential (ΔΨM). Various studies on immune cells and other cell models have demonstrated that an initial rise in ΔΨM occurs before or concurrently with the release of pro-apoptotic molecules and precedes caspase activation. This sequence of events ultimately leads to final membrane depolarization.^43^^,^^44^^,^^45^^,^^46^

To this aim, JC1 flow cytometric analysis was adopted to assess the ΔΨM of PBMCs and Jurkat over a 72-h exposure period, at 12 h intervals (Figures 9C–9F). The results showed significant changes in ΔΨM, as indicated by the MFI ratio normalized on CTR’s transmembrane potential of activated immune cells. The MV fraction exposure caused a significant initial hyperpolarization (*p* < 0.05 vs. PHA; Figures 9C–9F), followed within 48 h by a depolarization (*p* < 0.05 vs. PHA; Figures 9C–9F). The kinetics of mitochondrial membrane potential variation recorded in PBMCs and Jurkat exposed to MV faithfully reproduced the transmembrane potential pattern recorded in immune cells mitochondria pharmacologically exposed to high concentrations of DMSO, although in this latter experimental condition the ΔΨM recorded was significantly greater (*p* < 0.05; Figures 9C–9F). EIPA treatment mitigated the MV fraction effects (*p* < 0.05 vs. AEC ± LPS_MV_; Figures 9C–9F), further supporting the role of macropinocytosis in mediating the immunosuppressive mechanism. Of note, not only did EIPA significantly reduce the hyperpolarization-activated from both MV fractions (*p* < 0.05; Figures 9C–9F) but also brought the mean transmembrane potential back to levels like those recorded at 48 h in PBMCs and Jurkat after PHA activation (*p* > 0.05; Figures 9C–9F). The impairment of ΔΨM induced in immune cells from MV fraction internalization underscores the crucial link between alteration of mitochondrial membrane integrity and the late apoptosis documented from caspase 3/7 activation.

## Discussion

This study significantly advances the knowledge of the immune modulatory mechanisms governing AEC, cells belonging to a crucial amniotic layer that ensures successful pregnancy progression. At the same time, a novel intercellular communication strategy employed by AEC has been identified, which has promising biotechnological applications leveraging this perinatal stem cell source to be largely and readily available, ethically uncontentious, and immune-privileged.^47^^,^^48^^,^^49^^,^^50^^,^^51^

Indeed, AEC, the primary immunological barrier at the maternal-fetal interface, naturally produces a large amount of MVs containing immune bioactive components enabling dynamic communication with surrounding cells confirmed both *in vitro* and *in vivo*. The evidence of this intercellular dialogue *in vivo* is quite relevant to definitively support its key immune paracrine role and, at the same time, to exclude that the transfer of AEC-MV cargo is merely an *in vitro* artificial phenomenon.

Both *in vitro* and *in vivo* studies demonstrate that MV-mediated intercellular trafficking is highly efficient and primarily mediated via micropinocytosis, through which biocomponents are internalized into recipient cells. This mechanism was mainly validated by tracking donor mitochondrial internalization, strongly provided by mtDNA heteroplasmy *in vivo*. This MV transfer readily induced a response into recipient immune cells and organisms; however, mitochondrial integration into the host zebrafish larvae appears to be temporary. Indeed, approximately 90% of the transferred mitochondrial copies degraded within 48 h, indicating a limited duration of the transferred cargo’s functionality. This behavior contrasts with other multicellular contexts where long-term mtDNA integration occurs across genders and species.^38^

Of note, macropinocytosis emerges as the main mechanism driving AEC-derived MV internalization into immune cells. Indeed, the experiments revealed that intercellular trafficking of MV cargo and their biological action are almost completely prevented by EIPA supplementation, a potent inhibitor of macropinocytosis. This mechanism, constitutive in certain immune cells, may facilitate intercellular cargo transfer by reducing the need for a molecular complementarity between donor and recipient cells.^52^ This widely conserved process, that mediates not selective uptake of any solute or small particle, is regulated in a non-specific manner by extracellular environmental signals, without requiring any MV interactions with specific cell membrane receptors which could limit internalization.^53^

Apart from the straightforward intercellular transfer mechanism used for MV intercellular trafficking, AEC emerged as a privileged cell source for developing stem cell-mediated production of MV aimed to induce mitochondrial transfer. AEC are not only able to intrinsically encapsulate cargo of intracellular organelle within MV but do that with a high efficiency by considering the large amount of MV released and the easiness whereby can be taken up from recipient cells.^54^ Among the various bioactive components, the transfer of whole, healthy mitochondria to recipient cells has profound therapeutic implications for restoring tissue homeostasis.^55^^,^^56^ It may support, as recently demonstrated, the exogenous replacement of damaged mitochondria, thereby rescuing mitochondrial defects.^57^ The mitochondrial transfer mediated from AEC-MV, in addition, can potentially overcome the limited spontaneous mitochondrial transfer in tissues and organs.

Beyond their role in MV-mediated intercellular communication, AECs also regulate organelle biogenesis in response to inflammatory signals, such as those from bacterial invasion or tissue damage. Indeed, the lipidomic analysis revealed a distinctive lipid and organelle fingerprint in the AEC-derived secretomes, with the endoplasmic reticulum (ER) and mitochondria being the most represented. Notably, only the MV fraction contained ceramides, these lipids are pivotal in the formation and release of EVs and influence EV biogenesis by inducing spontaneous membrane invagination, which allows the formation of intraluminal vesicles (ILVs) within multivesicular bodies (MVBs).^58^^,^^59^ They also play a role in EV-mediated miRNA cargo segregation and transfer, suggesting they may contribute to the EV-mediated signaling in the AEC inflammatory context.^58^ In terms of mitochondria and ER, exposure to an inflammatory signal, such as LPS, alters the lipid profile of the MV, increasing certain PE lipid species while reducing polyunsaturated fatty acids and the concentrations of mitochondria and ER components. This behavior likely serve as a protective mechanism against the stress induced by the treatment, as previously observed in this model.^49^ The interplay between ER and mitochondria is further underscored by the role of mitochondria-associated ER membranes (MAMs), known to mediate critical functions in calcium homeostasis and the formation of the NLRP3 inflammasome.^60^^,^^61^ Given that MAMs are crucial for inflammasome assembly following TLR stimulation, the reduced mitochondrial fraction observed in response to LPS may reflect a cellular response to inflammation, where mitochondrial components are retained intracellularly to facilitate inflammasome formation.

Nonetheless, most of the MV-mediated immunomodulatory properties studied *in vitro* resulted unaffected by the status of the donor AEC and were highly conserved across species. A similar immunomodulatory influence of ovine AEC-derived MV cargo was observed on both PBMCs and human Jurkat cells. Notably, ovine MV internalization specifically exerted a strong inhibitory influence by inducing apoptosis in both immune cell models. Although the specific bioactive MV component initiating immune programmed cell death remains to be identified, it is evident that the internalization of MV cargo triggers apoptosis via caspase activation. This mechanism may recognize an upstream activation event in the modulation of mitochondrial transmembrane potential that starts with an early hyperpolarization to then hesitates into a final depolarization.^43^^,^^44^^,^^45^^,^^46^ Based on this effect, a key role for the transferred AEC-derived mitochondria could be hypothesized. Trafficking of mitochondrial cargo has been previously demonstrated to be involved in various cell death modalities and is viewed as both a sensor and amplifier of cell death.^62^ Denser mitochondria may lead to detrimental ROS amplification and increased cytochrome *c* production when cells are exposed to harsh environments, resulting in insufficient cell clearance and cell apoptosis.^63^ On the other hand, it has been shown that acquired mitochondria can promote an increase in endogenous mtDNA concentration.^64^ The regulatory mechanism exerted by MV derived from AEC cannot solely based in immune cells on the activation of programmed cell death, but it also involves other overlapping mechanisms. Amongst these, the inhibition of the intracellular NFAT pathway may play a role in this context.^39^^,^^40^^,^^41^^,^^42^^,^^65^^,^^66^^,^^67^ The beneficial local influence of PBMCs death may not be limited to hindering the inflammatory response, but it is also responsible for pro-regenerative tissue mechanisms. Human PBMCs apoptosis has been recognized have a role in the early phase of tissue healing resulting in the local release of favorable lipid mediators, proteins, and EVs, mainly exosomes and apoptotic bodies, responsible for enhancing angiogenesis and pro-regenerative processes.^65^^,^^68^^,^^69^

When the MV trafficking effect was tested on the *in vivo* model, for the first time, a donor-dependent action was demonstrated thus highlighting the diverse biogenesis and packaging characterizing the MV of AEC under intrinsic or activated conditions. This diverse immunomodulatory behavior of MV may be the consequence of the higher complexity of the model or the different immune target cells recorded. Whatever the reason, the exposure to MV during the early inflammatory phase of larvae zebrafish induced by caudal fin cut accelerated the local infiltration of innate immune cells. However, the transgenic immune cell recruitment was enhanced when the MV transfer involved secretomes collected from LPS-stimulated AECs, leading to a more robust and faster immune cell presence at the wound site.

The present results highlight the potent immunomodulatory action of AEC exerted not only through their MV-free fraction, as confirmed for several perinatal cells,^49^ but also through the MV one. The merit of the present research is to pose the first elements into understanding the convergent immune control exerted by the two paracrine fractions. The soluble component released by AEC enhances its inhibitory role on PBMCs activation in response to inflammatory insults,^49^^,^^70^^,^^71^^,^^72^ which in the present study are mimicked by LPS. Indeed, as shown in previous studies, these soluble components significantly increase in response to LPS, such as bioactive PGE2 and/or AREG that are released on demand by stressed cells to potentiated cell immunomodulatory control.^49^^,^^72^ Of note, most of these soluble molecules are intrinsically released by AEC but they become mainly active on immune cells at concentrations higher than those released under basal conditions.^49^ However, AEC can equally exert a steady immunomodulatory action at the maternal-fetal interface, by using the MV cargo transfer to shut down the immune cell activation.

### Limitations of the study

This study provides valuable insights into the immune-modulatory potential of AEC-derived MVs, yet some limitations warrant consideration. Specifically, the use of immortalized human AECs, while instrumental for achieving consistent and reproducible results, does not fully replicate the biological and physiological properties of naive ovine AECs. These freshly isolated cells better represent the primary immune context relevant to the *in vivo* environment. The human cells tested here are not exact analogues of the ovine AECs, and this distinction may influence certain translational applications. Addressing these differences through direct comparative studies would enhance the robustness and clinical relevance of the findings. Additionally, while macrophage phagocytosis is a plausible mechanism for MV uptake, this study focused primarily on the biological effects of mitochondrial transfer. Preliminary findings suggest that macrophages and other immune cells may play a significant role in managing transferred mitochondria, warranting further exploration using zebrafish larvae and iAEC to clarify these pathways.

Overall, this study underscores the significant potential of AEC as a prompt and largely available stem cell source for organelle production, transfer, and transplantation. The collected evidence makes key challenges clear such as MV cargo donor selection, transfer mechanism feasibility, and immunomodulatory control mechanisms. These insights offer several practical advantages for developing innovative therapies minimizing the culture passages and cell manipulation. Finally, the present research not only advances our understanding of AEC biology but also provides concrete evidence for the clinical exploitation of their innate immune control, which is physiologically maintained at the maternal-fetal interface from early embryonic development to delivery.

## Resource availability

### Lead contact

Further information should be directed and will be fulfilled by the lead contact, Dr. Giuseppe Prencipe (gprencipe@unite.it).

### Materials availability

This study did not generate new unique reagents or materials.

### Data and code availability

• All data reported in this paper will be shared by the lead contact upon request.
• This paper does not report the original code.
• Any additional information required to reanalyze the data reported in this working paper is available from the lead contact upon request.

## Acknowledgments

This project has received funding from the 10.13039/501100007601European Union’s Horizon 2020 research and innovation program under the Marie Skłodowska-Curie grant agreement No 955685 (www.p4fit.eu). A.C. was funded by 10.13039/501100003407MiUR within the framework of PON “Ricerca e Innovazione” 2014–2020 (PON R&I). Azione IV 0.4 “Dottorati e contratti di ricerca su tematiche dell’innovazione” in attuazione del DM 1062 del 10 agosto 2021. Project title: Emerging mechanisms on EMT-mediating stemness and tissue regeneration. H.A.S. acknowledges financial support from 10.13039/501100005075UMCG Research Funds. The facilities and expertise of HiLIPID and Professor Reijo Käkelä, at the University of Helsinki, supported by 10.13039/100015735HiLIFE, are gratefully acknowledged for the lipidomic analysis. Graphical abstract and some figures were created using BioRender.com.

## Author contributions

Conceptualization, A.C.-V., G.P., M.M., and B.B.; methodology, A.C.-V., G.P., and L.S.; formal analysis, A.C.-V., G.P., A.H., L.S., and A.I.; investigation, A.C.-V., G.P., A.P., A.C., M.P., and L.S.; writing – original draft, A.C.-V., G.P., A.C., L.S., and B.B.; writing – review and editing, A.C.-V., G.P., M.P., V.R., M.M., and B.B.; supervision, H.A.S., M.M., and B.B.; project administration, V.R. and B.B.; funding acquisition, H.A.S., V.R., and B.B.

## Declaration of interests

The authors declare no competing interests.

## STAR★Methods

### Key resources table

**Table:** 

REAGENT or RESOURCE	SOURCE	IDENTIFIER
**Antibodies**		

Mouse monoclonal anti-CD3	Invitrogen, Thermo Fisher Scientific	Cat# 16-0037-81; RRID: AB_468854
Mouse monoclonal anti-CD28	Invitrogen, Thermo Fisher Scientific	Cat# 16-0289-81; RRID: AB_468926
Rat monoclonal anti-CD44	Invitrogen, Thermo Fisher Scientific	Cat# MA4400; RRID: AB_223517
Rabbit polyclonal anti-CNX	MyBiosource	Cat# MBS806773
Mouse monoclonal anti-CD63	Biolegend	Cat# 353030; RRID: AB_2687004
Rabbit polyclonal anti-TOMM40	MyBiosource	Cat# MBS5400122
Rabbit monoclonal anti-HSP60	Cell Signaling	Cat# 56831; RRID: AB_2799521
Mouse monoclonal anit-HSP60	Enzo Life Sciences	Cat# ADI-SPA-806-F; RRID: AB_11177888

**Chemicals, peptides, and recombinant proteins**		

LPS	Sigma-Aldrich	Cat# L2637
PHA-L	Invitrogen	Cat# 00-4977-93
Penicillin-Streptomycin	Lonza	Cat# DE 17-602E
Amphotericin B	Euroclone	Cat# ECM0009D
L-Glutamine	Euroclone	Cat# ECB300D
P4	Sigma-Aldrich	Cat# P8783
Ficoll-Paque PLUS	Cytiva	Cat# GE17-1440-02
Fluoromount	Sigma-Aldrich	Cat# F4680
PBS	Sigma-Aldrich	Cat# P3813
PBS with Ca^+2^ and Mg^+^	Sigma-Aldrich	Cat# D8662
BSA	Sigma-Aldrich	Cat# P3813
Trypsin-EDTA 0.25%	Sigma-Aldrich	Cat# T4049
Quick Start™ Bradford 1x Dye Reagent	Bio-Rad Laboratories	Cat# 5000205
4X Laemmli Sample buffer	Bio-Rad Laboratories	Cat# 1610747
Phosphatase Inhibitor	SERVA Electrophoresis GmbH	Cat# 39055
Protease Inhibitor Cocktails	Sigma-Aldrich	Cat# P2714
Precast gel with a density gradient of 4–15%	Bio-Rad Laboratories	Cat# 4568083
Nitrocellulose membranes	Bio-Rad Laboratories	Cat# 1620145
Trans-Blot Turbo 5x Transfer Buffer	Bio-Rad Laboratories	Cat# 10026938
Every blot blocking solution	Bio-Rad Laboratories	Cat# 12010020
1X TBS 1% Casein Blocker	Bio-Rad Laboratories	Cat# 1610782
ClarityMax ECL reagent	Bio-Rad Laboratories	Cat# 1705062
Normocin™	InvivoGen	Cat#ANT-NR-2
Blasticidin	InvivoGen	Cat#ANT-BL-1
Zeocin®	InvivoGen	Cat#ANT-ZN-1
DMSO	Sigma-Aldrich	Cat# D8418
QUANTI-luc^TM^, Luciferase Detection Reagent	InvivoGen	Cat# REP-QLC4LG1
TRICAINE PHARMAQ 1000 mg/g	PHARMAQ AS	Cat# 104497045
EIPA	Selleck Chemicals	Cat# S9849
Mitotracker Red	Invitrogen	Cat# M7513
Mitotracker Green	Invitrogen	Cat# M7514
JC1	Invitrogen	Cat# T3168
Hoechst	Thermo Fisher Scientific	Cat# 62249
DAPI	Sigma-Aldrich	Cat# D9542

**Deposited data**		

Amniotic Epithelial Cell’s Secretome Phospholipid Analysis	This paper; Figshare	https://doi.org/10.6084/m9.figshare.28194215.v1

**Critical commercial assays**		

CellTiter96 Aqueous One Solution Cell Proliferation Assay	Promega	Cat# G3582
CellEventTM Caspase-3/7 Green Flow Cytometry Assay	Thermo Fisher Scientific	Cat# C10427
Quick-DNA Miniprep Plus kit	Zymo Research Corporation	Cat# D4068
SYBR Lo-ROX kit	Bioline	Cat# BIO-94050

**Oligonucleotides**		

Ovis aries mtDNA forward primer	In house	CTAGGCCTGTCCCTACTGGT
Ovis aries mtDNA reverse primer	In house	GCAAGCTGTGAAGTGTGGTG

**Experimental models**		

AEC	University of Teramo	N/A
iAEC	Nihon University School of Medicine	N/A
PBMCs	University of Teramo	N/A
Jurkat-Lucia^TM^ NFAT cells	InvivoGen	Cat# jktl-nfat-cd28
Zebrafish transgenic larvae (*Tg(lysC:dsRed2*)	University of Teramo	N/A

**Software and algorithms**		

ImageJ 1.53k	NIH	https://imagej.nih.gov/ij/
GraphPad Prism 10	Graphpad	https://www.graphpad.com
Biorender	Biorender	https://www.biorender.com
IZON Control Suite 1.4	IZON	https://www.izon.com
MassHunter Qualitative Navigator	Agilent	https://www.agilent.com
NIS-Element software 4.40	Nikon	https://www.microscope.healthcare.nikon.com/products/software/nis-elements
CytExpert SRT	Beckman	https://www.beckman.it/flow-cytometry/research-flow-cytometers/cytoflex/software
LIMSA	–	https://doi.org/10.1021/ac061390w

**Other**		

FBS	Gibco	Cat# 10270106
α MEM	Euroclone	Cat# ECM0850L
IMDM	Thermo Fisher Scientific	Cat# 12440053
DMEM	Euroclone	Cat# ECM0728L

### Experimental model and study participant details

#### Ethical statement

The amniotic cells used for the present research have been isolated from adult pregnant sheep. No authorization is needed for the collection and use of such cells since amniotic membranes are waste products of animals slaughtered for food consumption.

The zebrafish developmental stage at 72–120 hpf did not fall into the regulatory frameworks dealing with animal experimentation, and all the experiments complied with “Directive 2010/63/EU of the European Parliament and of the Council of 22 September 2010 on the protection of animals used for scientific purposes” and with the Italian law “D.Lgs n. 26 4 marzo 2014 Attuazione della direttiva 2010/63/UE sulla protezione degli animali utilizzati a fini scientifici”.

#### Isolation and culture of ovine amniotic-derived AEC

AEC was isolated from AM isolated from at least 3 pregnant Appenninica breed sheeps at the middle stage of gestation (25–30 cm in fetus length),^73^ to avoid the activation of epithelial-mesenchymal transition (EMT) at the term stage.^73^^,^^74^^,^^75^ Briefly, the uterus wall was carefully opened to isolate sterile amnios. Then, AM was mechanically peeled from the chorion with the aid of a stereomicroscope, and the tissue was dissected into 2–3 cm fragments. The AM pieces were washed in phosphate-buffered saline (Ca^+2^, Mg^+2^ free) (PBS; #P3813; Sigma-Aldrich, St. Louis, MO, USA) before isolating AEC by incubating them under gentle agitation in 0.25% Trypsin-EDTA (#T4049; Sigma-Aldrich, St. Louis, MO, USA) at 38.5°C for 40 min. The enzymatic digestion was blocked by adding 10% Fetal Bovine Serum (FBS; #10270106; Gibco, Thermo Fisher Scientific, Waltham, MA, USA) before filtration on a 40 μm pore membrane. The isolated cells were pelleted by centrifugation at 500*g* for 10 min before counting the vital ones using LUNA-II Automated Cell Counter (Logos Biosystems Inc., Gyeonggi-do, Korea) with the aid of trypan blue staining.

AEC were subsequently seeded at 10^4^ cells/well on 6 well plates using α-Minimum Essential Medium Eagle (α-MEM; #ECM0850L; Euroclone S.p.A., Milan, Italy), supplemented with 10% inactivated FBS (#10270106; Gibco, Thermo Fisher Scientific, Waltham, MA, USA), 1% L-Glutamine (#ECB300D; Euroclone S.p.A., Milan, Italy), 1% amphotericin B (#EUM0009D; Euroclone S.p.A., Milan, Italy), and 1% penicillin/streptomycin (#DE17-602E; Lonza, Basel, Switzerland) (hereafter referred as a complete medium), with 25 μM Progesterone (P_4_) (4 pregnene-3,20-dione, P_4_, #P8783; Sigma-Aldrich Corp., St. Louis, MI, USA) to prevent Epithelial-to Mesenchymal Transition (EMT).^76^^,^^77^ The cells were maintained at 38.5°C with 5% CO_2_ upon 70% confluence had been reached. Then, AEC were trypsinized (0.05% Trypsin-EDTA) for further experimental procedures.

#### Isolation of ovine PBMCs

Ovine PBMCs were isolated through a density gradient centrifugation starting from 16 mL of fresh peripheral blood collected at the slaughterhouse (from at least 3 Appenninica breed sheeps). The gradient was built up with 12 mL Ficoll-Paque PLUS (#GE17-1440-02; Cytiva, Marlborough, MA, USA) following the manufacturer’s instructions. Following the procedures mentioned above, the cells were preserved by storing them in liquid nitrogen until needed for immunological tests.

#### Culture of Jurkat Reporter Cells

Jurkat-Lucia NFAT cells (#jktl-nfat-cd28; InvivoGen, Toulouse, France), are a sensitive report T cell line that enables the measurement of NFAT activity via an NFAT-inducible Lucia luciferase construct. Cells were cultured in Iscove’s Modified Dulbecco’s Medium (IMDM; #12440053; Gibco, Thermo Fisher Scientific, Waltham, MA, USA) supplemented with 10% heat-inactivated FBS (#10270-106; Gibco, Thermo Fisher Scientific, Waltham, MA, USA), 100 μg/mL penicillin/streptomycin (#DE17-602E; Lonza, Basel, Switzerland), 100 μg/mL Normocin (#ANT-NR-2; InvivoGen, Toulouse, France), 5 μg/mL Blasticidin (#ANT-BL-1; InvivoGen, Toulouse, France) and 100 μg/mL Zeocin (#ANT-ZN-1; InvivoGen, Toulouse, France) and passaged every 2 days until the 3^rd^ passage to maintain the cellular density within the suggested threshold at 37°C under 5% CO2 atmosphere.

#### iAEC cell culture

iAEC cell line (immortalized human AEC expressing mitochondria-targeted turboRFP) was kindly donated by the Toshio Miki Lab at Nihon University School of Medicine, Tokyo, Japan. Cells were cultured in High Glucose DMEM (High Glucose DMEM; #ECM0728L; Euroclone S.p.A., Milan, Italy), supplemented with 10% inactivated FBS (#10270106; Gibco, Thermo Fisher Scientific, Waltham, MA, USA), 1% L-Glutamine (#ECB300D; Euroclone S.p.A., Milan, Italy), and 1% penicillin/streptomycin (#DE17-602E; Lonza, Basel, Switzerland) (hereafter referred as a complete medium), and passaged every 2 days at 37°C under 5% CO2 atmosphere.

#### Zebrafish

The zebrafish transgenic lines *Tg(mpx:GFP)* and *Tg(lysC:dsRed2*) used in the experiments were obtained from the University of Teramo facility (protocol number n. 4236). Adult fish were kept in a circulating system tank (Tecniplast S.p.a., Buguggiate, Italy). The tank temperature was generally maintained between 27°C and 28°C, the pH at 7 ± 0.2, the conductivity at 500 ± 100 μS/cm, and the lighting conditions in the room were 14:10h (light: dark). Animals were fed twice a day with live food (*Artemia salina*) and supplemented with ZEBRAFEED (Sparos, Olhão, Portugal). Zebrafish eggs were collected from timed pairwise spawning using sloping breeding tanks (Tecniplast S.p.a., Buguggiate, Italy). Immediately after spawning, which was initiated by morning light, the eggs were collected, rinsed and unfertilized eggs or injured embryos were eliminated. At least 147 embryos were maintained at 27°C in a 100mm glass Petri dish in dilution water (DW) until the experimental time (72 hpf).

### Method details

#### CM production from amniotic-derived AEC/iAEC

CM production was started once freshly isolated AEC/iAEC reached 70% confluence on a 6-well plate (died cells never exceed 1–2%). Then, the cells were preconditioned in serum-free medium (αMEM +1% penicillin/streptomycin +1% Amphotericin B + 1% L-Glutamine) for 4h. According to previous studies in other cellular models^78^ and AEC,^49^^,^^77^^,^^79^ some of them were exposed to the inflammatory LPS stimulus (1 μg/mL, #L2637; Sigma-Aldrich, St. Louis, MO, USA) for 1h. Afterward, all the AEC groups (CTR and LPS) were washed twice with serum-free medium and cultured in the same for 24 h before CM collection. Subsequently, the AEC-derived CM were centrifuged at 500g for 10 min and the supernatants were stored at −80°C.

#### MV isolation and physical characterization

To isolate the MV fraction from the previously collected CM, ultracentrifugation at 25,000g for 5 min at 4°C was performed (Optima XPN-90, Beckman Coulter Life Sciences, Brea, CA, USA). The supernatant was removed and the pellet containing the MV fraction was resuspended in a complete medium or DW for subsequent experiments.

The particle size distribution and concentration of CM, purified MV, and MV-free fractions were assessed using the Exoid TRPS measurement system (iZON, Lyon, France) and analyzed with the Izon Control Suite Software (version 1.4). Samples were diluted 50 times in an electrolyte buffer and analyzed with NP150 and NP250 nanopores, stretched to 47 mm by applying 300 Pa pressure and 700 mV voltage. To determine particle concentration, an average of 500 particles per sample were counted and compared against size and concentration reference calibration particles (iZON, Lyon, France).

#### Lipidomic analysis

Lipids were extracted from whole secretome, MV-free, and MV fractions according to Folch et al.^80^ and dissolved in chloroform/methanol 1:2 (v/v). Internal standards [SPLASH internal standard mixture and Ceramide (Cer) 18:1; O2/17:0, both from Merck] were added before analysis with LC-MS/MS as described before.^81^ In brief, an aliquot of 7 μL of the extract was injected into the Agilent 1290 Infinity HPLC (Agilent Technologies, Santa Clara, CA) for chromatographic separation in a gradient mode with a Luna Omega C18 100 Å (50 × 2.1 mm, 1.6 μm) column (Phenomenex) using an acetonitrile/water/isopropanol-based solvent system.^82^ The lipid species were detected using lipid class-specific precursor ion and neutral loss detection modes with Agilent 6490 Triple Quad LC/MS with iFunnel Technology, as previously described.^81^ Mass spectra were processed using MassHunter Qualitative Navigator software (Agilent). The lipid species were identified based on their molecular and class-specific fragment ions and quantified utilizing the internal standards and LIMSA software.^83^ Lipid data are expressed as μmol/mg of total cell protein, and lipid species are marked as follows: [sum of acyl chain carbons]: [sum of acyl chain double bonds] (e.g., PC 36:1).

#### Pinocytosis investigation

To investigate the mechanism involved in mediating the mitochondrial transfer of MV-derived cargoes (AEC_MV_), the following treatments were performed.

(1) Inhibition of macropinocytosis using 50 μM EIPA (#S9849, Selleck Chemicals, Houston, Sylvanfield Dr, United States) during the:
  • 12/24-h incubation with AEC ± LPS_MV_ and AEC±LPS_MV-free_ fractions for mitochondrial transfer assessment.
  • 48-h incubation with AEC ± LPS_MV_ and/or AEC±LPS_MV-free_ fractions for proliferation, NFAT activation, and late apoptosis assays.
  • 72-h incubation with AEC ± LPS_MV_ for the evaluation of mitochondrial membrane potential.
(2) Inhibition of CD44 activity using 10 μg/mL Anti-CD44 antibody (#MA4400, Invitrogen, Thermo Fisher Scientific, Waltham, MA, USA) during the:
  • 12-h incubation with AEC ± LPS_MV_ and AEC±LPS_MV-free_ fractions for mitochondrial transfer assessment.
  • 48-h incubation with AEC ± LPS_MV_ and/or AEC±LPS_MV-free_ fractions for proliferation, NFAT activation, and late apoptosis assays.

#### Flow cytometry

For MV characterization, the following antibodies were utilized: CNX (1:100; #MBS806773, MyBioSource, San Diego, CA, USA), CD63 (1:100; #353030, BioLegend, San Diego, CA, USA), TOMM40 (1:100; #MBS5400122, MyBioSource, San Diego, CA, USA), and HSP60 (1:100; #56831, Cell Signaling Technology, Danvers, MA, USA). Additionally, MitoTracker Red detection (500 nM; #M7513, Invitrogen, Thermo Fisher Scientific, Waltham, MA, USA), JC1 (2 μM; #T3168, Invitrogen, Thermo Fisher Scientific, Waltham, MA, USA) analysis, and late apoptosis detection (CellEvent Caspase-3/7 Green Flow Cytometry Assay Kit, #C10427, Invitrogen, Thermo Fisher Scientific, Waltham, MA, USA) were performed. Samples were thoroughly washed with DPBS (#D8662; Sigma-Aldrich, St. Louis, MO, USA) and subsequently suspended in DPBS containing 0.05% BSA (#A3059, Sigma-Aldrich, St. Louis, MO, USA). Flow cytometry analyses were conducted using a CytoFLEX SRT system (Beckman Coulter, Brea, Calif., USA), analyzing a total of 10,000 events per sample. Excitation was performed using 405 nm, 488 nm, and 561 nm lasers, detecting fluorescence in the V450, B525, B690, and Y585 channels. Fluorescence intensity was represented on a standard logarithmic scale. Data analysis and visualization were performed using CytExpert SRT software (version 1.2.10004, Beckman Coulter, Brea, Calif., USA).

#### Western blotting

Total protein was extracted from each sample using RIPA lysis buffer (#R0278, Sigma-Aldrich, St. Louis, MO, USA) supplemented with Phosphatase Inhibitor (#39055; SERVA Electrophoresis GmbH, Heidelberg, Germany) and Protease Inhibitor Cocktails (#P2714; Sigma-Aldrich, St. Louis, MO, USA) diluted according to manufacturer’s instruction. Samples were placed on ice for 30 min and then centrifuged at 12,000 x g for 12 min at 4°C. The supernatant was collected, and 5 μL was used to determine protein concentration with Quick Start Bradford 1x Dye Reagent (#5000205; Bio-Rad Laboratories, Milan, Italy). Proteins were separated by running a precast gel with a density gradient of 4–15% (#4568083; mini-PROTEAN precast gel, Bio-Rad Laboratories, Milan, Italy), and subsequently transferred to nitrocellulose membranes (#1620145; Bio-Rad Laboratories, Milan, Italy) using Trans-Blot Turbo 5x Transfer Buffer (#10026938; Bio-Rad Laboratories, Milan, Italy) and Trans-Blot Turbo Transfer (Bio-Rad Laboratories, Milan, Italy). Membranes were then incubated with EveryBlot blocking solution (#12010020; Bio-Rad Laboratories, Milan, Italy) for 5 min. The primary antibody for HSP60 (1:500, ADI-SPA-806-F, Enzo Life Sciences, Inc., NY, USA), was diluted in 1X TBS 1% Casein Blocker (#1610782; Bio-Rad Laboratories, Milan, Italy) and incubated overnight at 4°C. The specific anti-mouse secondary HRP antibody (1:10.000 Cell Signaling Technology, Danvers, MA, USA) was diluted in the solution mentioned above and incubated for 1h at RT. ClarityMax ECL reagent (#1705062; Bio-Rad Laboratories, Milan, Italy) was used to visualize the target protein and the chemiluminescent signal was detected using the ChemiDoc MP Imaging System (Bio-Rad Laboratories, Milan, Italy). Densitometric analysis was performed using ImageJ (ImageJ 1.53k, NIH, Bethesda, MD, USA). Data were normalized on total protein content via Stain-Free technology (Bio-Rad Laboratories, Milan, Italy).

#### Immunofluorescence

To detect mitochondria, PBMCs’ mitochondria and MV’s mitochondria were stained with MitoTracker Green FM (200 nM; #M7514, Invitrogen, Thermo Fisher Scientific, Waltham, MA, USA) or MitoTracker Red (500 nM; #M7513, Invitrogen, Thermo Fisher Scientific, Waltham, MA, USA), respectively, following the manufacturer’s instructions. Specifically, for MV staining, the samples were incubated with MitoTracker Red for 30 min at 37°C. After incubation, the samples were centrifuged at 25.000 g for 5 min, and then washed twice with cell culture medium, with centrifugation at 25.000*g* for 5 min after each wash. The resulting pellet was resuspended in a cell culture medium or DW for PBMCs, Jurkat Reporter cells, and zebrafish larvae exposure, respectively. PBMCs and Jurkat Reporter cells were exposed to stained AEC ± LPS_MV_ for 12 h, while zebrafish larvae were visualized from 6 h post-exposure. Subsequently, the samples were analyzed using flow cytometry or imaged with confocal microscopy. For confocal imaging, PBMCs’ nuclei were stained with Hoechst (diluted 1:1000 in PBS; #62249, Thermo Fisher Scientific, Waltham, MA, USA); and zebrafish larvae nuclei were stained with DAPI (diluted 1:100 in DW; #D9542; Sigma-Aldrich, St. Louis, MO, USA) for 10 min at RT. Zebrafish larvae *Tg (mpx:GFP)* were used to visualize neutrophils with green fluorescence. Images were acquired under a Nikon Ar1 laser confocal scanning microscope (Nikon, Düsseldorf, Germany) equipped with the NIS-Element software 4.40 (Nikon, Düsseldorf, Germany).

#### DNA extraction

To quantify the interspecific transfer of ovine MVs, DNA was extracted from a pool of 1x10^6^ Jurkat Reporter cells and 10 zebrafish larvae for each condition, 24 h post-treatment, and from 1mL of AEC_MV_ to serve as a positive control.

DNA extraction was performed using the Quick-DNA Miniprep Plus kit (#D4068, Zymo Research Corporation, Irvine, CA, USA) following the manufacturer’s instructions. For zebrafish larvae, the initial lysis step was slightly modified, to improve DNA yield: samples were homogenized in Cell Lysis Buffer (provided by the DNA extraction kit) supplemented with Proteinase K using an automatic pestle, and then heated on a thermoblock at 55°C for 30 min. The extracted DNA was quantified spectrophotometrically via NanoDrop 2000c (Thermo Fisher Scientific, Waltham, MA, USA). 1 μL of each DNA sample was loaded into the spectrophotometer, and absorbance at 230, 260, and 280 nm was measured to provide sample concentration (ng/μL) and purity.

#### Real-time qPCR

To evaluate the copy numbers representing the amount of ovine mitochondrial DNA (mtDNA) transferred after MVs’ mitochondria internalization in Jurkat Reporter Cells and zebrafish larvae 24- and/or 48-h post-treatment, a Real-Time qPCR was performed. qPCR was carried out in triplicate using the SensiFAST SYBR Lo-ROX kit (#BIO-94050, Bioline, Cincinnati, OH, USA), on a (QuantStudio 3 Real-Time PCR; Applied Biosystems, Thermo Fisher Scientific, Waltham, MA, USA), according to the manufacturer’s instructions. The amplification reaction protocol was as follows: 95°C for 3 min, followed by 40 cycles at 95°C for 5 s and 60°C for 30 s.

#### Quantification of ovine mtDNA copy number in zebrafish and Jurkat

After DNA extraction, the mtDNA copy number was measured. Specific forward and reverse primers for ovine mtDNA were designed based on the Ovis aries mtDNA sequence using the NCBI Primer-BLAST tool and used at a final concentration of (0.5 pmol/μL). Absolute quantification of ovine mtDNA (targeting the region between 10103 and 10273 nt) was performed using real-time qPCR, with amplification results converted to mtDNA copy numbers via a standard curve. The sequences of primers used in Real-Time qPCR are the following: forward primer 5′ CTAGGCCTGTCCCTACTGGT 3’ (annealing temperature: 60.03°C); reverse primer 5′ GCAAGCTGTGAAGTGTGGTG 3’ (annealing temperature: 59.97°C).

#### PBMCs activation test

The immunomodulatory effect of different MV and MV-free fractions (AEC ± LPS) was verified using the MTS PBMCs activation test. In detail, PBMCs were seeded in 96-well plates at the concentration of 2 × 10^5^ cells, activated with 10 μg/mL of phytohemagglutinin (PHA-L; #00-4977-93; Invitrogen, Thermo Fisher Scientific, Waltham, MA, USA) and cultured for 48h with or without AEC ± LPS_MV_ and AEC±LPS_MV-free_ fractions. Then, PBMCs proliferation was assessed by using the “CellTiter 96 Aqueous One Solution Cell Proliferation Assay” (#G3582; Promega, Corporation, Madison, WI, USA) following the manufacturer’s instruction protocol. Data were normalized on PHA-activated PBMCs.

#### NFAT pathway activation

To test the MV and MV-free fractions’ effects on specific intracellular immune cell signaling pathways controlling proliferation, Jurkat-Lucia NFAT cells were used. Reporter Jurkat cells amplified for 3 passages were seeded at a concentration of 3 × 10^5^ cells on a 96-well high binding plate (#9018; Corning, NY, USA), previously coated with a CD3/CD28 (2 μg/mL, #16-0037-81 and #16-0289-81, respectively; Invitrogen, Thermo Fisher Scientific, Waltham, MA, USA). The culture was maintained for 48h in the presence or absence of AEC ± LPS_MV_ and AEC±LPS_MV-free_ fractions. The evaluation of the mean luminescence value was assessed, after the supplementation of the QUANTI-luc, Luciferase Detection Reagent (#REP-QLC4LG1; InvivoGen, Toulouse, France), by using EnSpire Multimode Plate Reader (PerkinElmer, Waltham, MA, USA). Data were normalized on the CD3/CD28 stimulated Jurkat.

#### MV Fraction’s’ immunomodulatory properties on transgenic zebrafish

To assess the effects of the MV fraction *in vivo*, *Tg(lysC:dsRed2)* zebrafish larvae at 72 hpf were used. Firstly, the larvae were anesthetized with TRICAINE PHARMAQ (PHARMAQ AS, Norway) at 0.02% and with a sharp scalpel, the distal tip of the tailfin was dissected to induce acute inflammation. Then, the larvae were exposed to MV fractions derived from AEC ± LPS for 6h. Fluorescence intensity was detected at the Nikon Eclipse Ti in time-lapse and quantified using the plugin RGB measure on ImageJ software (ImageJ 1.53k, NIH, Bethesda, MD, USA).

### Quantification and statistical analysis

At least three biological replicates were employed for AEC (fetus), PBMCs (animals), and zebrafish samples to verify inter-experimental variability. Each experiment was replicated three times to evaluate intra-experimental variability. The exact values of n (representing the sample size or individual animals) and the statistical significance are provided in the captions of the figures. Mean ± S.D. represented the data, with D'Agostino and Pearson tests utilized to assess normal distribution. One-way ANOVA and two tailored t-tests were employed for comparing normally distributed data, followed by Tukey post hoc analyses (GraphPad Prism 10, San Diego, CA, USA). ALL, ∗, ^Δ^, ^▪^, ∗∗, ∗∗∗, ∗∗∗∗, Statistically significant values between the different studied groups (*p* < 0.01, *p* < 0.05, *p* < 0.05, *p* < 0.05, *p* < 0.01, *p* < 0.001, and *p* < 0.0001, respectively).
