# Supplementary material for: Amniotic epithelial Cell microvesicles uptake inhibits PBMCs and Jurkat cells activation by inducing mitochondria-dependent apoptosis
Source: iScience. 2025 Jan 18;28(2):111830. doi: 10.1016/j.isci.2025.111830 (PMC11834128; doi:10.1016/j.isci.2025.111830)
Supplement: Document S1. Figure S1 [file mmc1.pdf]

iScience, Volume *28*

Supplemental information

Amniotic epithelial Cell microvesicles uptake inhibits PBMCs and Jurkat cells activation by inducing mitochondria-dependent apoptosis

Adrián Cerveró-Varona, Giuseppe Prencipe, Alessia Peserico, Angelo Canciello, Andrew H. House, Hélder A. Santos, Monia Perugini, Ludovica Sulcanese, Chika Takano, Toshio Miki, Annamaria Iannetta, Valentina Russo, Mauro Mattioli, and Barbara Barboni

Supplemental Information

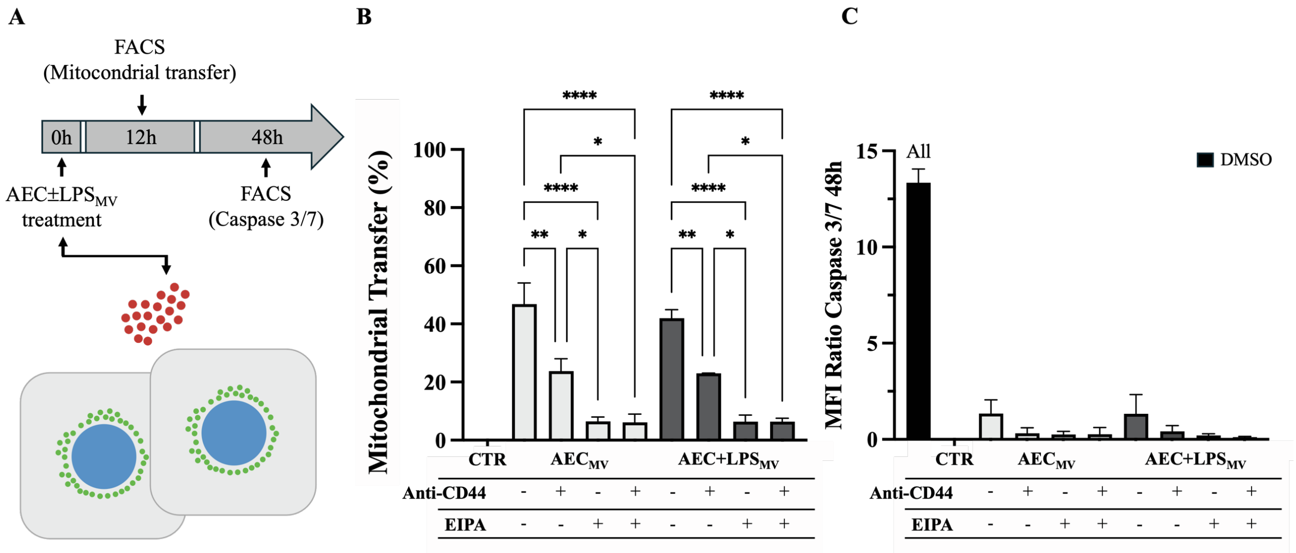

Figure S1. Mitochondrial Tracking Confirmed Safe Uptake of AEC-Derived MV Cargo by AECs Without Triggering Apoptosis, related to Figure 4*.*

*(**A**) Experimental design for B-C. (**B**) Flow cytometric analysis confirmed the internalization of MV-derived mitochondria (labeled with MitoTracker Red) in AEC with and without LPS treatment, in the presence or absence of CD44 and EIPA. Data are expressed as the percentage of cells positive for MitoTracker Red. The untreated AEC served as the control (CTR). (**C**) Flow cytometric analysis of Caspase 3/7 activity was performed on AEC treated as described above for 48 hours. No apoptosis was detected under any condition. Data are presented as the mean fluorescence intensity (MFI) ratio over the negative CTR.*

*Data (mean ± SD) represent 3 independent sets of experiments (n = at least 3 biological replicates in each group per set; each biological replicate assayed in at least 3 technical replicates). ALL, *, **, and **** Statistically significant values between the different studied groups (p<0.0001, p<0.05, p<0.01, and p<0.0001, respectively).*
